# Homozygous *ARHGEF2* mutation causes intellectual disability and midbrain-hindbrain malformation

**DOI:** 10.1371/journal.pgen.1006746

**Published:** 2017-04-28

**Authors:** Ethiraj Ravindran, Hao Hu, Scott A. Yuzwa, Luis R. Hernandez-Miranda, Nadine Kraemer, Olaf Ninnemann, Luciana Musante, Eugen Boltshauser, Detlev Schindler, Angela Hübner, Hans-Christian Reinecker, Hans-Hilger Ropers, Carmen Birchmeier, Freda D. Miller, Thomas F. Wienker, Christoph Hübner, Angela M. Kaindl

**Affiliations:** 1 Institute of Cell Biology and Neurobiology, Charité University Medicine Berlin, Berlin, Germany; 2 Department of Pediatric Neurology, Charité University Medicine Berlin, Berlin, Germany; 3 Sozialpädiatrisches Zentrum (SPZ), Center for Chronic Sick Children, Charité University, Berlin, Germany; 4 Max Planck Institute for Molecular Genetics, Berlin, Germany; 5 Guangzhou Women and Children's Medical Center, Guangzhou, China; 6 Department of Molecular Genetics, University of Toronto, Toronto, Canada; 7 Program in Neurosciences & Mental Health, Hospital for Sick Children, Toronto, Canada; 8 Max-Delbrück-Center for Molecular Medicine, Berlin, Germany; 9 Department of Pediatric Neurology, University Children's Hospital of Zurich, Zurich, Switzerland; 10 Department of Human Genetics, University of Würzburg, Würzburg, Germany; 11 Pediatrics, University Hospital, Technical University Dresden, Dresden, Germany; 12 Gastrointestinal Unit and Center for the Study of Inflammatory Bowel Disease, Massachusetts General Hospital, Harvard Medical School, Boston, Massachusetts, United States of America; University Medical Center, GERMANY

## Abstract

Mid-hindbrain malformations can occur during embryogenesis through a disturbance of transient and localized gene expression patterns within these distinct brain structures. Rho guanine nucleotide exchange factor (ARHGEF) family members are key for controlling the spatiotemporal activation of Rho GTPase, to modulate cytoskeleton dynamics, cell division, and cell migration. We identified, by means of whole exome sequencing, a homozygous frameshift mutation in the *ARHGEF2* as a cause of intellectual disability, a midbrain-hindbrain malformation, and mild microcephaly in a consanguineous pedigree of Kurdish-Turkish descent. We show that loss of *ARHGEF2* perturbs progenitor cell differentiation and that this is associated with a shift of mitotic spindle plane orientation, putatively favoring more symmetric divisions. The *ARHGEF2* mutation leads to reduction in the activation of the RhoA/ROCK/MLC pathway crucial for cell migration. We demonstrate that the human brain malformation is recapitulated in *Arhgef2* mutant mice and identify an aberrant migration of distinct components of the precerebellar system as a pathomechanism underlying the midbrain-hindbrain phenotype. Our results highlight the crucial function of ARHGEF2 in human brain development and identify a mutation in *ARHGEF2* as novel cause of a neurodevelopmental disorder.

## Author summary

During brain development, localized gene expression is crucial for the formation and function of specific brain regions. Various groups of proteins are known to regulate segmentation through controlled gene expression, among them, the Rho GTPase regulator family. In this study, we identified a frameshift mutation in the Rho guanine nucleotide exchange factor 2 gene (*ARHGEF2*) in two children presenting with intellectual disability, mild microcephaly, and a midbrain-hindbrain malformation. This phenotype is also observed in *Arhgef2* mutant mice, highlighting the importance of ARHGEF2 across development of distinct mammalian species. We show that loss of Arhgef2 affects neurogenesis and also cell migration. In addition, we extended the current knowledge of ARHGEF2 expression and its role in early central nervous system development, with special reference to the formation of the precerebellar system. In addition to extensive literature on ARHGEF2, we now provide evidence for its significant role in neuronal migration in brain development and link the gene to human neurodevelopmental disease.

## Introduction

Brain development depends on spatiotemporally controlled gene expression.[1–3] Alterations in the expression pattern of such genes can result in neurodevelopmental disorders by impinging on key processes such as neural progenitor specification, cell division, and differentiation and the migration of newly born neurons from their site of origin to their final destination within the brain.[4–6] The latter is crucial for the formation of specific brain structures.[7–9] Factors that control localized gene function include Rho GTPase regulators. Here, we present evidence that the loss of function of Rho guanine nucleotide exchange factor 2 (ARHGEF2) causes a human neurodevelopmental disorder characterized by intellectual disability, mild microcephaly, and midbrain-hindbrain malformation.

ARHGEF2 (synonym GEF-H1, murine Lfc) catalyzes the replacement of GDP to GTP bound to Rho-related proteins and thereby controls timing and localization of the activation of Rho GTPases such as RhoA.[10–14] ARHGEF2 connects microtubule and actin cytoskeleton dynamics.[14] In this context ARHGEF2 activity is reduced through microtubule binding and further controlled by upstream regulators.[15–25] ARHGEF2 is key for actin and microtubule reorganization and is required for mitotic spindle formation and orientation.[11] Inhibition of ARHGEF2 results in spindle disorientation and dysfunction, mitotic delay, accumulation of prometaphase cells, and further mitotic aberrations.[11, 22] In mouse neocortex, *Arhgef2* is expressed in neural precursor and immature neurons and regulates neurogenesis from cortical precursor cells.[24] *Arhgef2* down-regulation by shRNA keeps radial precursors cycling, potentially by disrupted spindle plane orientation, and thereby inhibits neurogenesis. In contrast, *Arhgef2* overexpression causes an increase of neurons in the cortical plate.[24–26] Arhgef2 also plays a role in neural tube closure by regulating morphogenetic movements.[27] Furthermore, Arhgef2 participates in the migration of non-neuronal cells and in Wnt-induced planar cell polarity, via the activation of RhoA.[28, 29] Although evidence for a central function of Arhgef2 in cytoskeletal dynamics and critical signal transduction pathways exists and other ARHGEF genes have been linked with neurological disease,[30–32] little is known about ARHGEF2 function in humans and no disease phenotype associated with this gene has been reported.

## Results and discussion

**Fig 1:**
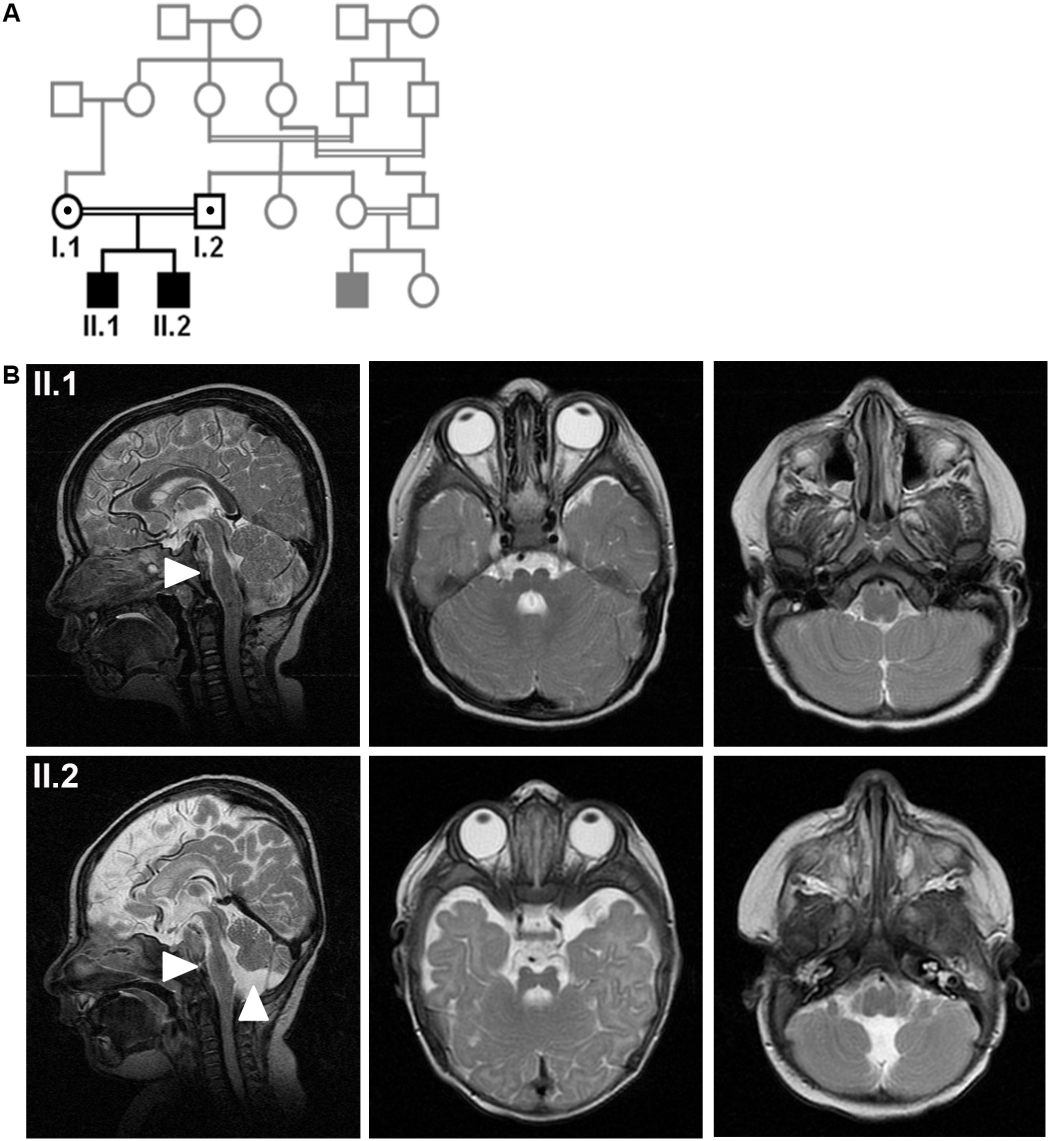
Phenotype of patients with midbrain-hindbrain malformation and intellectual disability. (**A**) The index patients are children (II.1, II.2) of healthy consanguineous Kurdish parents (first cousins) from Turkey. (**B**) Cranial MRI revealed microencephaly and hypoplasia of the pons and grooves (II.1, II.2) as well as hypoplasia of the cerebellum (II.2) or the caudal vermis (II.1). Arrow heads indicate hypoplastic pons (II.1, II.2) and cerebellum (II.2). Sagittal and axial T2 images.

We report that patients with a homozygous mutation in *ARHGEF2* develop intellectual disability, mild microcephaly, and midbrain-hindbrain malformations. Two affected children of healthy, consanguineous parents of Kurdish-Turkish descent were born at term without complications after an uneventful pregnancy (II.1; II.2, Fig 1A). At birth, mild congenital microcephaly with occipitofrontal head circumferences (OFC) of -1.95 (II.1) and -2.33 (II.2) SDS (standard deviation score) but normal weight and height were apparent (S1 and S2 Tables). In addition, the two boys variously displayed wide intermamillary distance, broad fingers, low posterior hairline, and facial dysmorphism with long philtrum, thin upper lip, high palate, downslanted palpebral eye fissures, long eyelashes, bilateral ptosis, and horizontal pendular nystagmus (S1 Table). Ophthalmological examination revealed congenital strabismus, astigmatism, amblyopia (II.2), optic disc pallor (II.2), abnormalities of the retinal pigment epithelium (II.2), and abnormal visual-evoked potentials further underlining optic nerve affection. Motor milestones were not severely delayed despite generalized muscular hypotonia observed in patient II.1 and weak tendon reflexes in both children. Both children stumbled frequently and had a disturbance of fine motor movements (S3 Table). Moderate intellectual disability (IQ <50) and severe developmental speech delay despite normal hearing capacity were diagnosed in both patients (S1 and S3 Tables). Cranial magnetic resonance imaging (MRI) revealed mild microencephaly, elongated midbrain, hypoplasia of the pons, ventral and dorsal longitudinal clefts (grooves) in pons and medulla, and inferior vermis hypoplasia (Fig 1B). The finding of grooves and cerebellar hypoplasia (in absence of the ‘dragonfly sign’ with hypotrophy of the cerebellar hemispheres rather than of the vermis) argued against the differential diagnosis of pontocerebellar hypoplasia. Results of routine blood tests, extensive metabolic work-up, chromosome analysis, and cardiac function assessment were normal.

**Fig 2:**
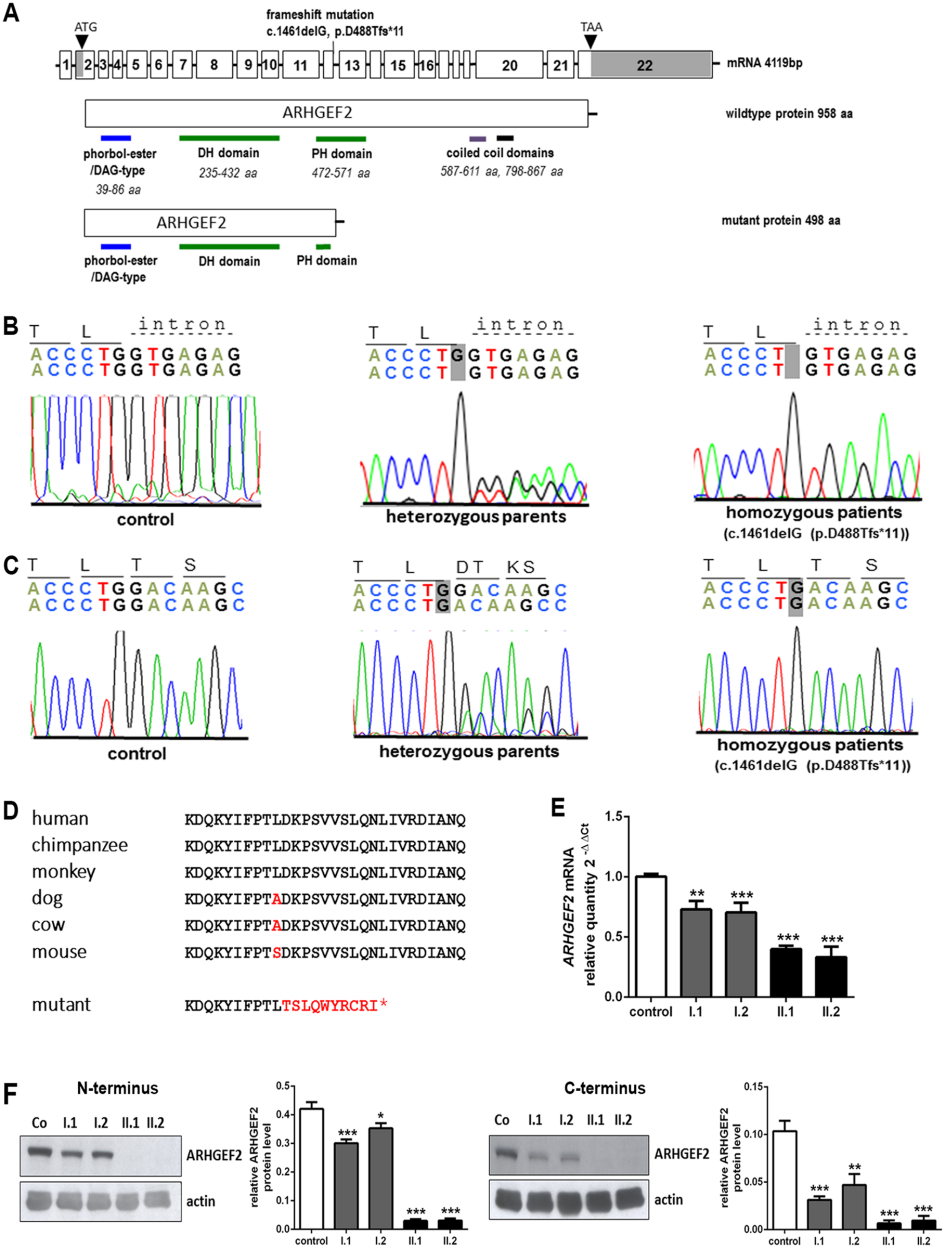
Homozygous *ARHGEF2* mutation in index patients. (**A**) By whole exome sequencing, the homozygous mutation c.1461delG (chr1:155,928,110delC, hg19) was identified in exon12-intron 12 boundary of the *ARHGEF2* (NM_004723.3). Pictogram representing ARHGEF2 protein domains (DH-DBL homology domain, PH-pleckstrin homology domain, phorbol-ester/Diacyl Glycerol-type zinc finger domain and coiled-coil domain) (**B**) Electropherogram depicting homozygous deletion of one base pair in the *ARHGEF2* in patient II.1, which is heterozygous in the healthy parents and normal in the healthy control. **(C)** This mutation leads to a transfer of the neighboring C in the coding region into the splice donor site, as shown by cDNA sequencing. This is predicted to cause a frameshift mutation (p.D448Tfs*498, NP_004714.2) and a null allele. (**D**) The mutation lies within a highly conserved region of the protein. (**E**) Quantitative real-time PCR revealed strongly reduced *ARHGEF2* transcript levels in patients and intermediate levels in heterozygous parents, as would be expected from partial nonsense-mediated mRNA decay (n = 3, One-way ANOVA). (**F**) ARHGEF2 protein levels were below detection levels on a protein immunoblot of lymphoblastoid cells from the affected subjects compared to a control, with intermediate levels in heterozygous parents (ARHGEF2 120 kDa, actin 43 kDa; n = 3, One-way ANOVA). *p<0.05, **p<0.01, ***p<0.001, Error bars represent ± SD.

To identify the genetic cause of this disease, we performed whole exome sequencing and bioinformatic analysis followed by Sanger sequencing in both affected individuals. We thereby identified a homozygous deletion of a single guanine (G) at the exon12-intron12 boundary (Fig 2A and 2B), causing deletion of a G at the exon12-exon13 transition from normally spliced cDNA and ultimately a frameshift mutation in the affected children (c.1461delG, NM_004723.3, Fig 2C). The mutation lies within a highly conserved region, results in predicted truncation of the protein (p.D488Tfs*11, NP_004714.2, Fig 2D), and segregates with the disease phenotype, i.e., was heterozygous in both parents and not observed in healthy controls. We failed to detect the deletion in various whole exome databases and did not detect biallelic *ARHGEF2* mutations in six further patients, as detailed in the methods chapter. In lymphoblastoid cell lines (LCLs), generated from the patients, *ARHGEF2* mRNA levels were decreased as can be expected by partial nonsense-mediated decay (Fig 2E, n = 3, One-way ANOVA). Moreover, ARHGEF2 protein levels were virtually absent in patient cells and decreased to intermediate levels in cells from the heterozygous parents (Fig 2F, n = 3, One-way ANOVA). The predicted truncated form of ARHGEF2 protein was below detection levels in patient LCLs (S1 Fig), indicating the total loss of ARHGEF2 in patient cells.

**Fig 3:**
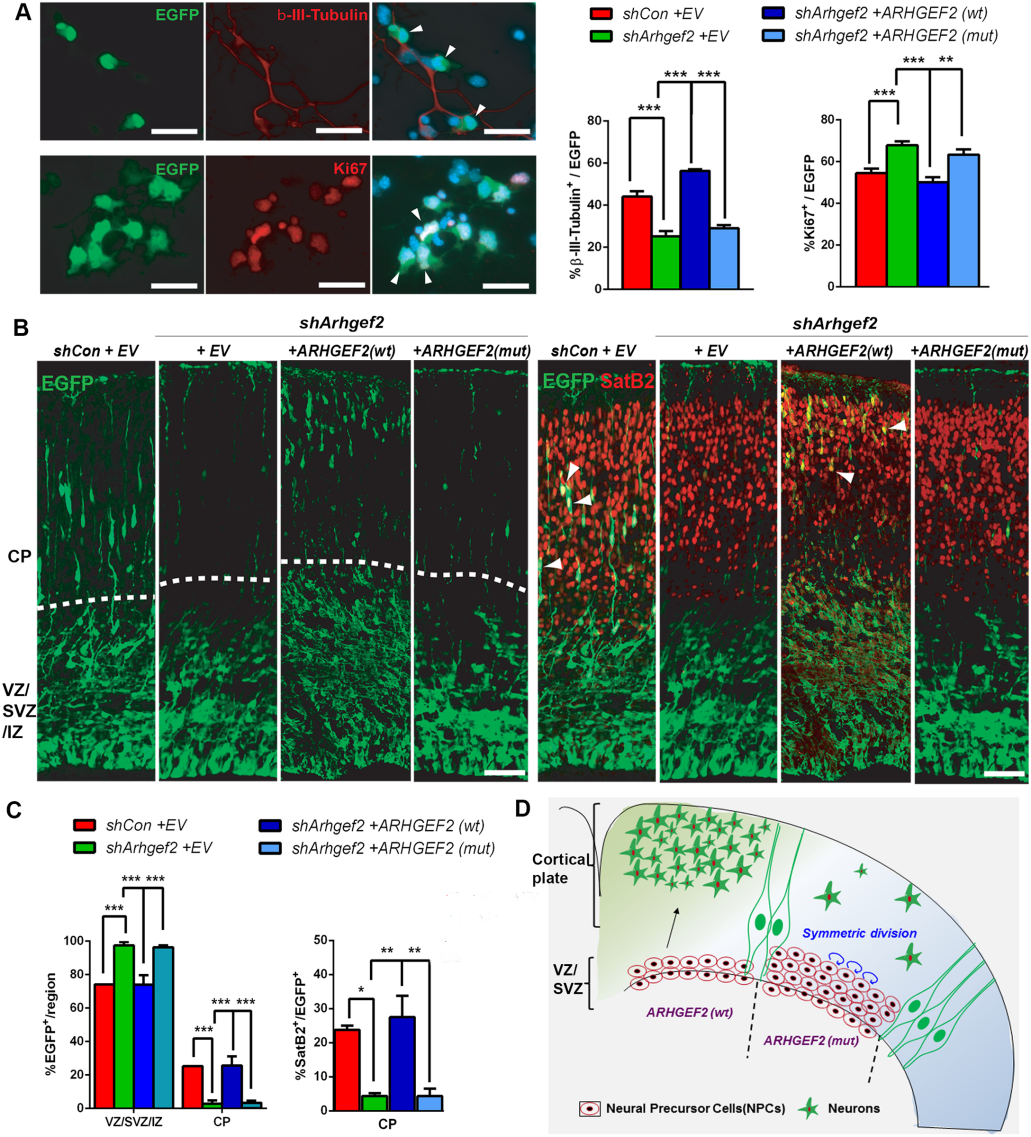
Loss of ARHGEF2 function inhibits neurogenesis and increases neural precursors *in vitro* and *in vivo*. **(A)** Cultured mouse cortical precursor cells were transfected with mouse *Arhgef2* shRNA (*shArhgef2*) or scramble shRNA (*shCon*), with a nuclear EGFP plasmid and with either an empty vector (EV), wildtype (wt) or mutant (mut) human *ARHGEF2*. Representative fluorescence micrographs of *shArhgef2* knockdown cultured precursors immunostained for EGFP (green), βIII-tubulin or Ki67 (red), and Hoechst 33258 nuclear staining (blue) 3 days after transfection; right panels show the merged images; arrowheads indicate double-positive cells. The *Arhgef2* shRNA-induced phenotype of decreased early neuronal marker βIII-tubulin-positive cells and increased proliferation marker Ki67-positive precursors is rescued by *ARHGEF2* (wt) but not *ARHGEF2* (mut) (n = 1378–1293 cells in 4 independent experiments, One-way ANOVA, scale bar 25 μm). **(B, C)** Quantification of EGFP positive cells and SatB2/EGFP double-positive cells (arrowheads) in E13.5 mouse cortices co-electroporated with plasmids specified above and analyzed 3 days later. Representative fluorescence micrographs of sections co-stained for SatB2 (new-born neurons) and EGFP (dotted line indicates boundary between CP and VZ/SVZ/IZ) (n = 3, One-way ANOVA, scale bar 50 μm). **(D)** Pictorial representation: ARHGEF2 favors neurogenesis and its downregulation/dysfunction inhibits neurogenesis by maintaining murine NPCs in cycling phase. *p<0.05, **p<0.01, ***p<0.001. Error bars represent ± S.E.M.

To evaluate the biological effects of the identified *ARHGEF2* mutation on brain development, we utilized both *in vitro* and *in vivo* approaches. We used a well-characterized cell culture model of E13 mouse neocortical cells recapitulating the timing of cortical precursor differentiation observed *in vivo*.[24, 33, 34] The large majority of these cells are actively dividing radial glial precursors positive for the neural progenitor marker nestin immediately following plating. These cells then differentiate to generate excitatory neurons (DIV1-3) and subsequently astrocytes and oligodendrocytes (DIV5-7). We demonstrated previously, using this model, that knockdown of *Arhgef2* causes a decrease in the proportion of βIII-tubulin positive neurons and a corresponding increase of cycling cortical precursors.[24] We thus expected the human *ARHGEF2* frameshift mutation to have a similar impact on neurogenesis, leading to an increase of cycling (and eventually apoptotic) precursors and a decrease of neurons generated during brain development. In a first series of experiments, we transfected murine cortical radial precursors with an *Arhgef2* shRNA plasmid and a nuclear EGFP reporter plasmid; the latter allows the visualization of transfected cells. As expected, *Arhgef2* knockdown significantly decreased the proportion of double positive cells for EGFP and early neuron marker βIII-tubulin, whereas it increased the proportion of EGFP and proliferation marker Ki67 double positive precursors (Fig 3A, n = 1378–1293 cells, One-way ANOVA). Next, we co-transfected cortical progenitors with the same combination of plasmids and an additional construct encoding for human wildtype *ARHGEF2*. In this condition, we observed no significant change in the proportion of EGFP and βIII-tubulin double positive cells or EGFP and Ki67 as compared to untransfected cells, demonstrating that wildtype ARHGEF2 rescues the phenotype produced by shRNA (Fig 3A). Last, we co-transfected cortical progenitors with *Arhgef2* shRNA, the EGFP reporter, and a construct encoding mutant *ARHGEF2* identified in the index patients. Here, the proportion of EGFP and βIII-tubulin double positive cells was reduced, whereas the number of EGFP and Ki67 remained increased (Fig 3A). We conclude that the phenotype produced by *Arhgef2* shRNA was rescued by coexpression of wildtype but not of mutant human *ARHGEF2*, consistent with the interpretation that the patient mutation is a loss-of-function mutation and that this *ARHGEF2* mutation impairs neurogenesis.

To further analyze whether the identified *ARHGEF2* loss-of function mutation affects brain development *in vivo* we *in utero* electroporated E13.5 mouse cortex with *Arhgef2* or *Con* (control) shRNA constructs along with wildtype or mutant human *ARHGEF2* plasmids as well as an EGFP vector. EGFP as a marker for electroporated cells and SatB2 as a marker for newborn neurons in murine cortices were quantified three days after electroporation. We identified a pronounced increase in the proportion of EGFP-positive cells in the ventricular, sub-ventricular, and intermediate zones (VZ/SVZ/IZ) and decreased proportions in the cortical plate (CP) of the *Arhgef2* shRNA electroporated mice, as compared to mice that were electroporated with the EGFP reporter only (Fig 3B and 3C). In line with our *in vitro* data, overexpression of wildtype, but not mutant, *ARHGEF2* was able to effectively rescue the cell distribution in the cortex induced by *Arhgef2* shRNA. Upon electroporation of *Arhgef2* shRNA, we additionally observed a dramatic reduction in the proportion of SatB2/EGFP double positive cells localized to the cortical plate. This is consistent with impaired neurogenesis described above in cultured cortical precursor cells. Overexpression of wildtype *ARHGEF2* was able to rescue this impairment of neurogenesis while mutant *ARHGEF2* was ineffective (Fig 3B and 3C, n = 3, One-way ANOVA). These data further confirm that the patient mutation in human *ARHGEF2* acts as a loss-of-function mutation also *in vivo*, results in dramatically decreased neurogenesis and is most probably disease-causative (Fig 3D).

**Fig 4:**
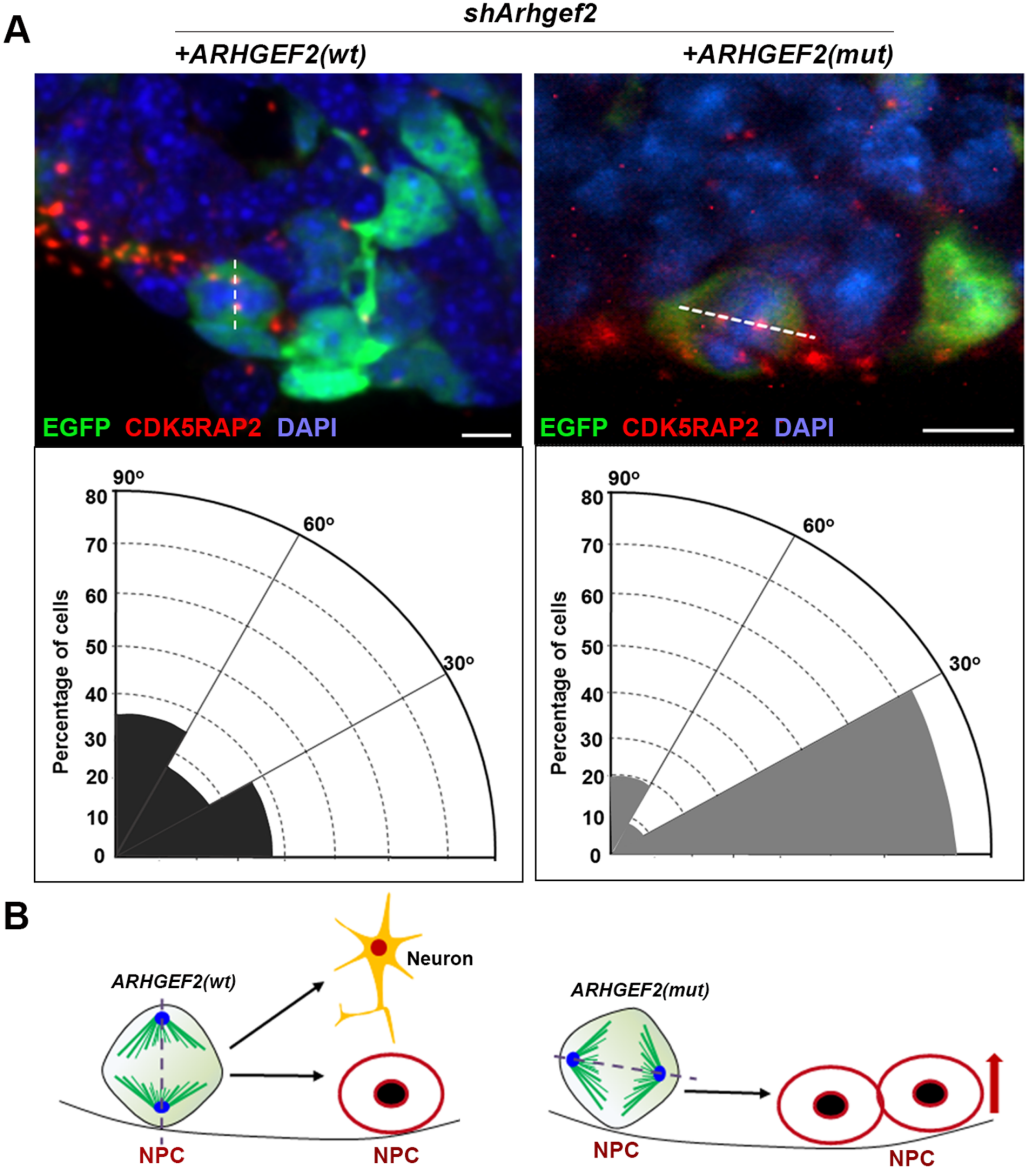
Mutant *ARHGEF2* favors self-renewing (symmetric) precursor proliferation. **(A)** Quantification of spindle plane orientation of dividing, metaphase and anaphase precursors transfected with EGFP (n = 68–70 cells, Student’s *t-test*, scale bar 20 μm). *Arhgef2* shRNA is associated with a more horizontal orientation of the spindle pole. Overexpression of *ARHGEF2* (wt), not *ARHGEF2* (mut), rescues the spindle orientation phenotype in dividing precursors. Representative fluorescence micrographs of sections stained with CDK5RAP2 (centrosomes), DAPI, and EGFP, indicating the plane of division (dotted line) and respective radial diagram. **(B)** Model indicating the plane of symmetry and cell fate decision in the developing brain. *p<0.05. Error bars represent ± S.E.M.

ARHGEF2 has been associated previously with spindle plane orientation,[11, 24] and the latter has been shown to play a crucial role for the fate of neuronal precursors, ‘deciding’ between self-renewal and differentiation.[35] To evaluate whether human mutant *ARHGEF2* alters neurogenesis by impairing mitotic spindle orientation, we performed *in utero* electroporation with the experimental setup described above and determined spindle plane orientation. We determined the angle between the ventricular surface and a line connecting centrosomes in metaphase/anaphase cells in brain sections. *Arhgef2* shRNA led to a significant shift of the spindle orientation towards a more horizontal orientation, characterizing putatively more symmetrically, self-renewing cell divisions. Again, overexpression of human wildtype but not mutant *ARHGEF2* was able to rescue significantly this phenotype (Fig 4A, n = 68–70 cells from 3–4 mice, One-way ANOVA). In the developing brain, ARHGEF2 reportedly controls the arrangement for symmetric or asymmetric cell divisions of apical progenitors through plane orientation.[24] Given our results from overexpressing mutant *ARHGEF2* in the mouse brain, we concluded that loss of ARHGEF2 activity inhibits neurogenesis by favoring more symmetric divisions of neocortical progenitors (Fig 4B).

**Fig 5:**
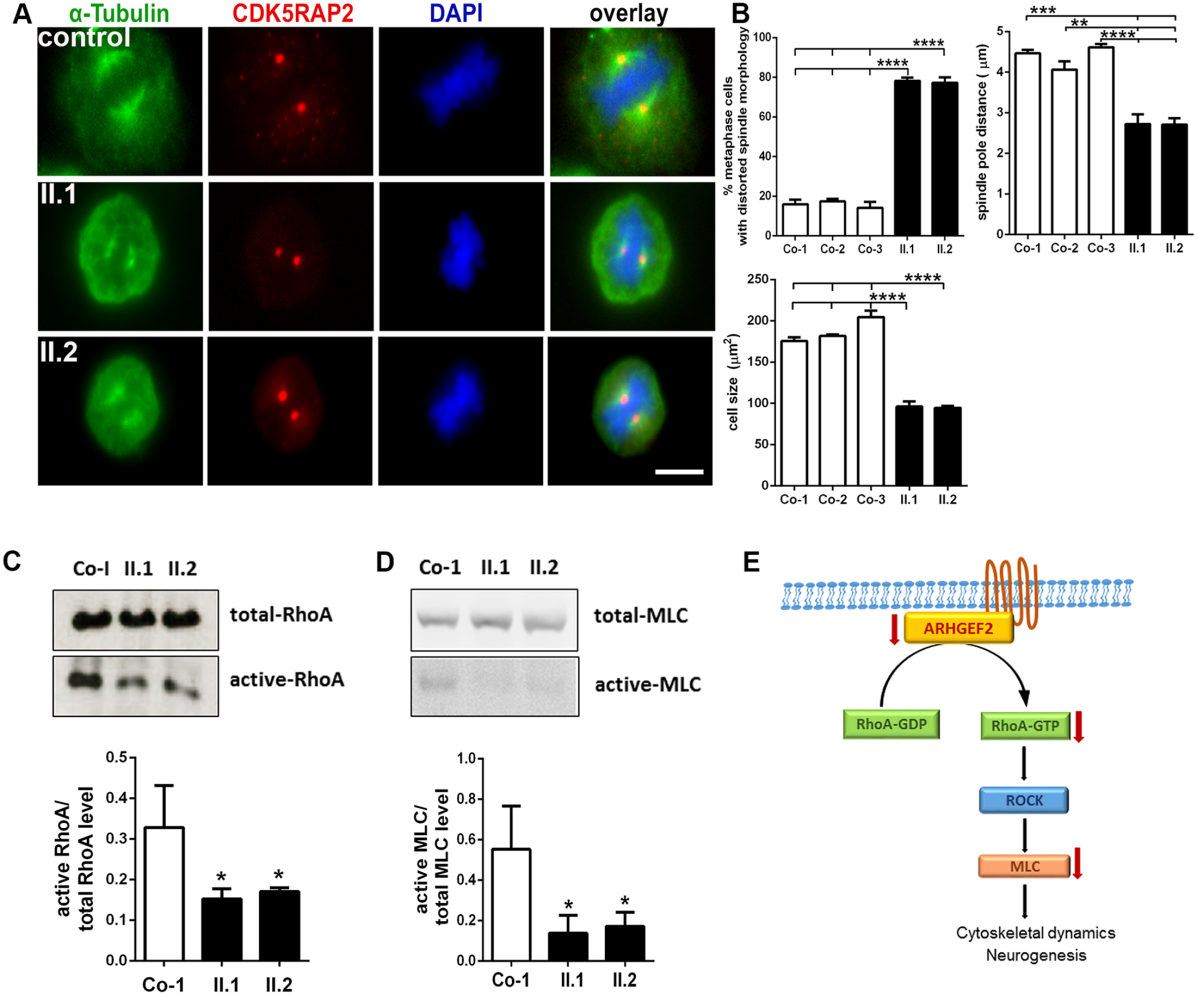
Mitotic spindle defects and reduced RhoA activation in LCLs of index patients with *ARHGEF2* mutation. (**A**) Abnormal spindle formation with an increase of abnormal misdirected spindles and broad, unfocused microtubules poles in immortalized patient II.1 and II.2 lymphocytes (LCLs, metaphase cells only of patient II.1 and II.2 are shown, centrosome marker CDK5RAP2 (red), spindle marker α-tubulin (green), DNA was stained with DAPI (blue)). CDK5RAP2 signals did not differ significantly between patient and control cells; this holds also true for the centrosome marker γ-tubulin (S3 Fig). (**B**) Quantification results underlining abnormal spindle morphology, decreased spindle pole distance, and reduced cell size of patient versus control LCLs (n = 100–200, One-way ANOVA, scale bar 5 μm): abnormal spindle morphology in patients (II.1 77%, II.2 78%) versus controls (Co-1 16%, Co-2 17%, Co-3 14%), decreased spindle pole distance in patients (II.1 2.70, II.2 2.72 μm) versus controls (Co-1 4.46, Co-2 4.05, Co-3 4.61 μm), and reduced cell size in patients (II.1 96.14, II.2 94.66 μm^2^) versus controls (Co-1 175.52, Co-2 181.74, Co-3 204.51 μm^2^). We observed no difference in size or proliferation of T-cells between *Arhgef2-/-* and *Arhgef2+/+* mice **(**S4 Fig**)**. (**C, D**) Reduced levels of active RhoA and active MLC in patient LCLs in protein immunoblots (n = 3, One-way ANOVA). (**E**) Pictogram of RhoA/ROCK/MLC pathway downstream of ARHGEF2, important for processes such as cytoskeletal dynamics and neurogenesis. *p<0.05, **p<0.01, ***p<0.001, ****p<0.0001. Error bars represent ± SD.

To further substantiate the effect of a loss of ARHGEF2 functions in human tissues and other cell types, we analyzed cell cycle apparatus in LCLs from our index patients and healthy controls. We detected an abnormal morphology of the mitotic spindle apparatus, decreased spindle pole distance, and decreased cell size in LCLs derived from the patients compared to controls, significantly (Fig 5A and 5B, n = 100–200 cells, One-way ANOVA). There was, however, no cell cycle defect, increased radiosensitivity, nor abnormal centrosome ‘morphology’ in the patient LCLs (S2 and S3 Figs). We concluded that regulation of the mitotic spindle apparatus is a primary function of ARHGEF2 in the cell cycle also in human tissues and other cell types.

ARHGEF2 regulates various cellular processes through activation of Rho family GTPases, specifically RhoA and its downstream effectors, such as RhoA/ROCK/MLC pathway members.[12, 22, 28, 36, 37] Specifically, RhoA has been implicated in the regulation of neurogenesis and planar cell divisions.[38, 39] We thus analyzed the activity of RhoA and its immediate downstream effectors in patient and control LCLs. We detected a significant reduction of active RhoA and, consistently, reduced levels of active phospho-myosin light chain (MLC) in the LCLs of affected individuals with an *ARHGEF2* mutation compared to healthy individuals (Fig 5C–5E, n = 3, One-way ANOVA). This indicates that the RhoA/ROCK/MLC pathway is most likely impaired in humans with the *ARHGEF2* mutation.

**Fig 6:**
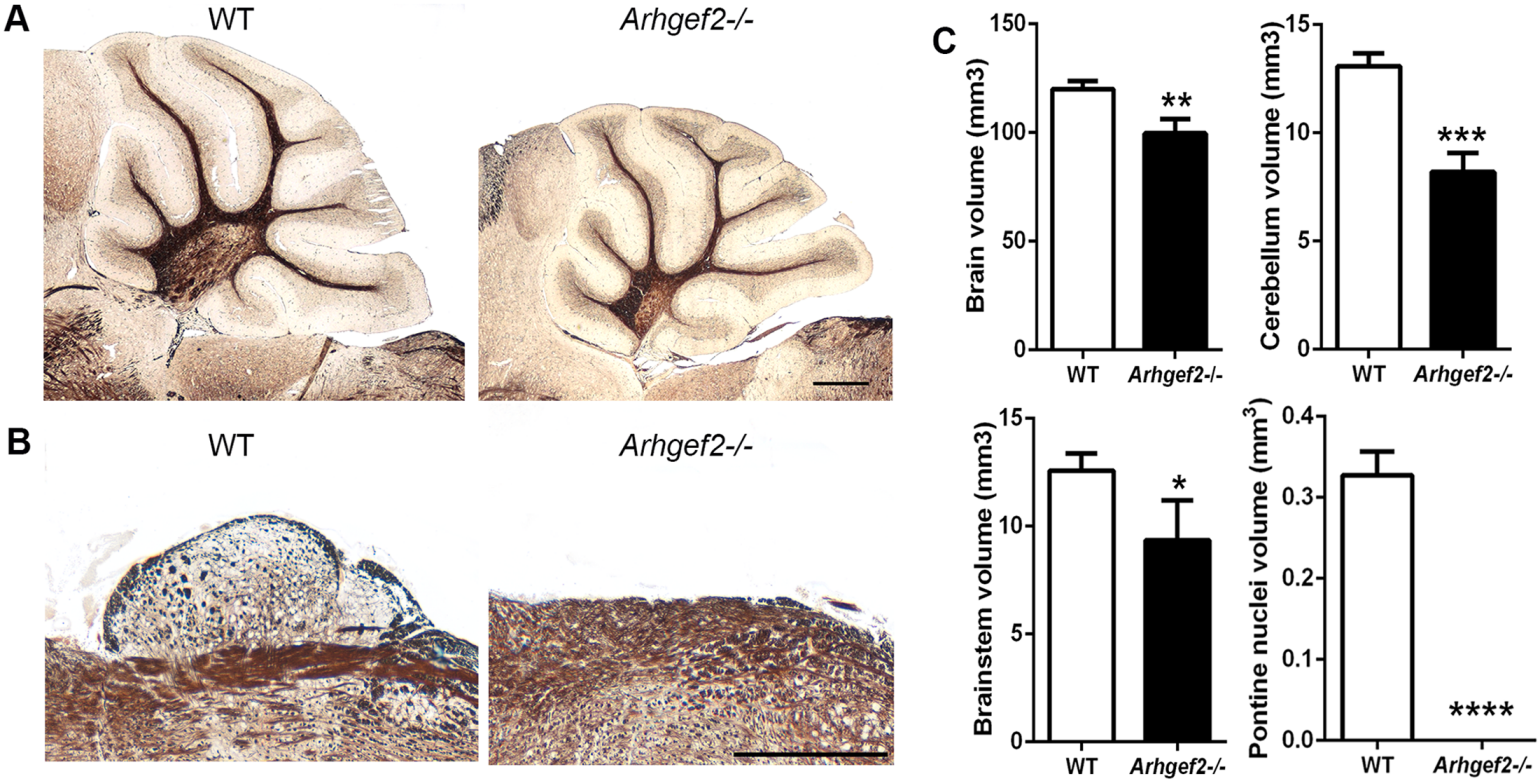
*Arhgef2-/-* mutant mice display microencephaly, cerebellar hypotrophy and lack pontine nuclei. **(A, B)** Reduced cerebellar size and lack of pontine nuclei in adult *Arhgef2* mutant mice compared to wild type littermates (Gallyas staining, DIC images, scale bar 500 μm). **(C)** Quantification underlines significant reduction of whole brain, cerebellum, brain stem, and pontine nuclei volume in mutant versus wild type brains (n = 3–4, Student’s *t-test*; *p<0.05, **p<0.01, ***p<0.001, ****p<0.0001. Error bars represent ± SD).

Previous studies showed that *Arhgef2* is expressed in the neural tube of mice from embryonic day 11 on and maintained at high levels during brain development.[24] Our *in situ* hybridization studies on embryonic mice showed strong expression of *Arhgef2* transcripts in the neuroepithelium of telencephalon, diencephalon, and rhombencephalon of E11 mice (S5 Fig). At the time of birth, *Arhgef2* is maintained in the germinal zones of both the neocortex and the cerebellum, as well as in the pontine gray nuclei (PGN) (S5 Fig). To further corroborate the pathogenicity of the identified *ARHGEF2* mutation in brain development, we analyzed the phenotype of *Arhgef2* deficient mice.[40] In adult *Arhgef2* mutant mice, we observed a significant reduction in volume of the total brain size (referred to as microencephaly), the cerebellum and the brainstem, as well as the striking absence of the pontine nuclei **(**Fig 6, n = 3–4, Student’s *t-test***)**. We concluded that the phenotypes observed in the nervous system of *Arhgef2* deficient mice recapitulate largely those malformations observed in the index patients. Our results showed a clear correspondence between regions displaying high levels of *Arhgef2* expression and those pathologically affected in the index patients with *ARHGEF2* mutation.

To gain further insight in the function of *ARHGEF2* in brain development, we set out to analyze the cerebral cortex, midbrain, cerebellum, and hindbrain of *Arhgef2* mutant mice. First, no drastic anatomical abnormality was found in the neocortex of *Arhgef2* mutant mice, as assessed by the measurement of its volume, thickness, and surface area (S6 Fig). Though it should be noted that there was a trend towards a reduction in the analyzed parameters in the mutant mice, which could contribute to the mild microcephaly phenotype observed in *Arhgef2* mutant mice. Similarly, no major alteration on the distribution of neurons in the cortical layers was found in *Arhgef2* mutant mice (S6 Fig). Next, we analyzed the integrity of midbrain structures by immunostaining the inferior and superior colliculus with antibodies against the transcription factor FoxP2. This revealed no significant alteration in development of midbrain structures in *Arhgef2* mutant mice (S7 Fig).

**Fig 7:**
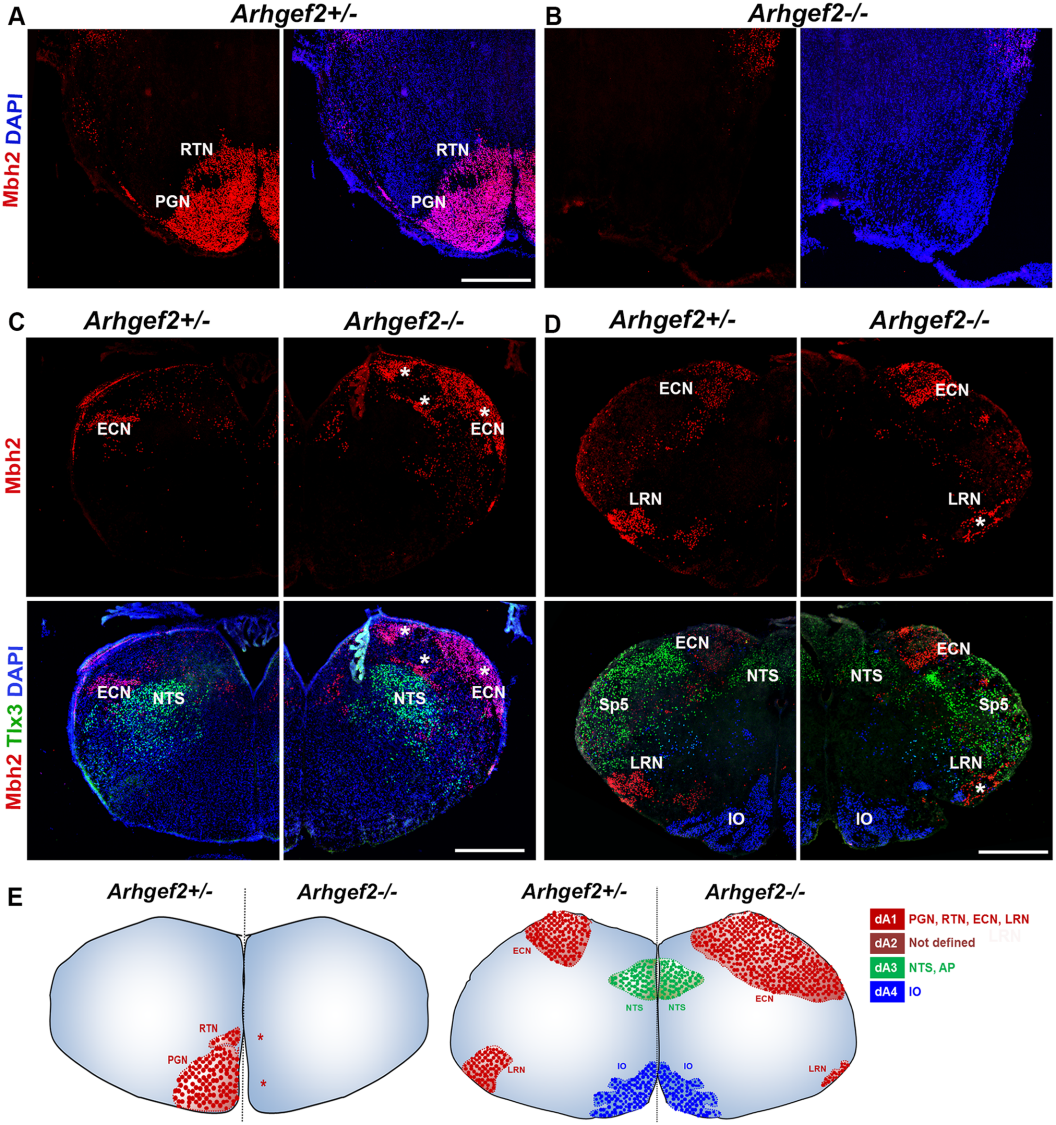
Loss of Arhgef2 affects the formation of precerebellar nuclei of dA1 derivatives. Representative fluorescence micrographs of coronal hindbrain sections of *Arhgef2-/-* and *Arhgef2+/-* (used as controls) mice, stained with precerebellar neuronal marker Mbh2 (red), Tlx3 (green), and DAPI (n = 3, scale bar 300 μm). **(A, B)** *Arhgef2-/-* mouse brains lack PGN and RTN present in *Arhgef2+/-* mouse brains. **(C)** In the rostral medulla of *Arhgef2-/-* mice, the ECN is abnormally enlarged and distributed (indicated by stars) when compared to *Arhgef2+/-* mice. **(D)** The LRN in the caudal medulla is significantly reduced in size (indicated by stars) in *Arhgef2-/-* mice compared to *Arhgef2+/-* mice, whereas the IO and NTS are unaffected. **(E)** Pictogram depicting the abnormal formation of dA1-derived precerebellar nuclei (red) and normal formation of dA3 (green) and dA4 (blue) nuclei in the *Arhgef2-/-* mice brain in comparison to the *Arhgef2+/-* control condition. Abbreviations: PGN-pontine gray nuclei, RTN-reticulotegmental nuclei, ECN-external cuneate nuclei, LRN-lateral reticular nuclei, IO-inferior olivary nuclei, NTS-nucleus of the solitary tract, Sp5-spinal trigeminal nuclei, AP-area postrema, dA-class A neurons.

**Fig 8:**
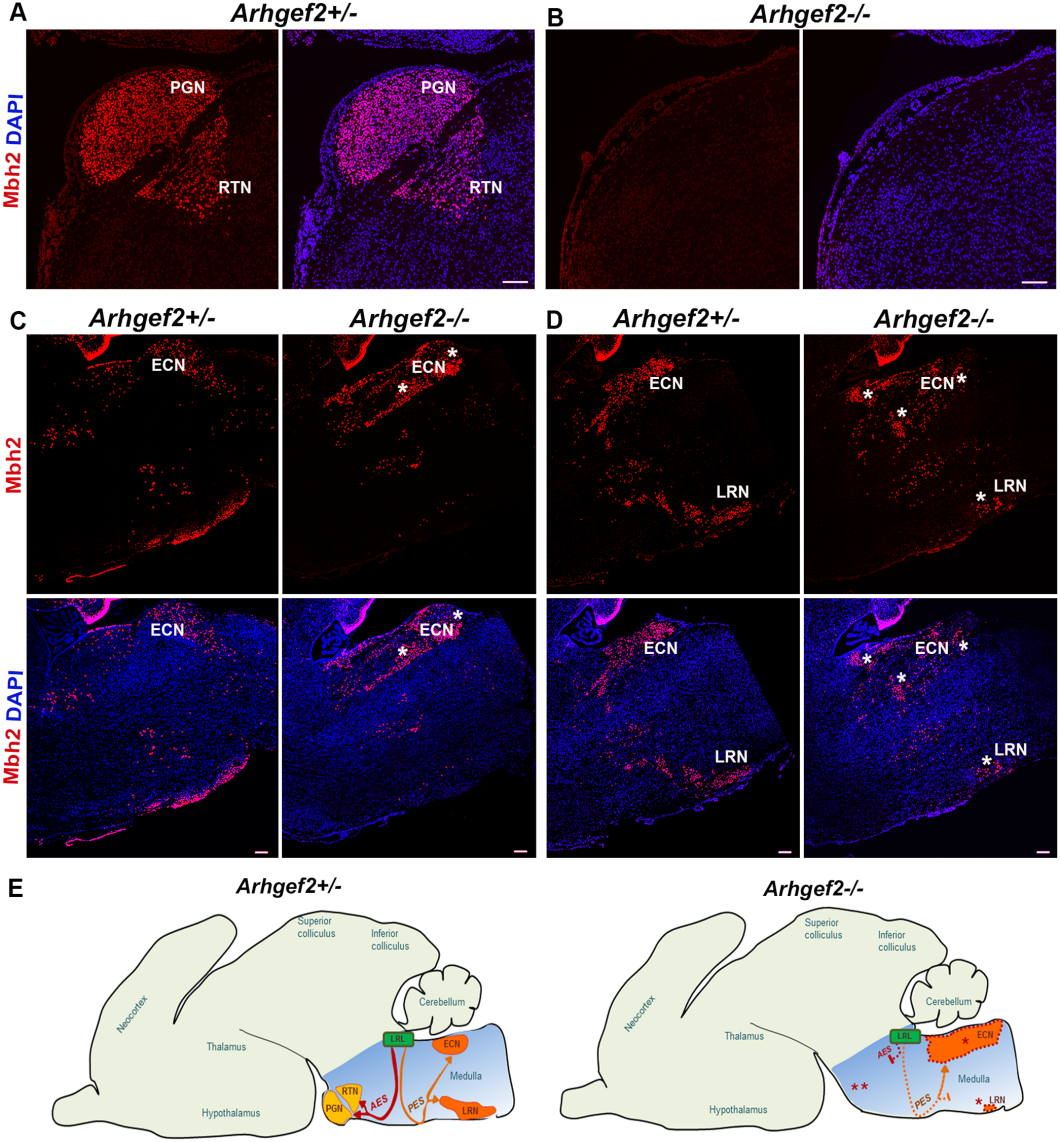
*Arhgef2-/-* mutant mice exhibit perturbed migration of precerebellar neurons. Sagittal hindbrain sections of *Arhgef2-/-* and *Arhgef2+/-* mice stained for precerebellar neurons with Mbh2 in red (n = 3, scale bar 100 μm, nuclei stained with DAPI (blue)). **(A, B)** PGN and RTN formed through AES migratory stream is absent in *Arhgef2-/-* and present in *Arhgef2+/-* mouse brains. **(C, D)** The disturbed PES migratory stream in the *Arhgef2-/-* mice is associated with the formation of enlarged ECN and smaller LRN compared to those in *Arhgef2+/-* mice. **(E)** Pictogram illustrating the normal formation of PGN and RTN via the AES migratory stream (red) and LRN and ECN via the PES migratory stream (orange) from the lower rhombic lip (LRL) in the mouse brain. In the *Arhgef2-/-* brain, AES and PES streams are disturbed (represented by dotted lines), culminating in abnormal migration and accumulation of precerebellar neurons. Abbreviations: PGN-pontine gray nuclei, RTN-reticulotegmental nuclei, ECN-external cuneate nuclei, LRN-lateral reticular nuclei, LRL-lower rhombic lip.

To address the cerebellar phenotype observed in *Arhgef2* mutant mice and patients, we measured the size of Purkinje cells and the thickness of the cerebellar molecular layer in *Arhgef2* mutant and control mice. Our analysis revealed no significant difference in these parameters between wildtype and mutants (S8 Fig). This led us to hypothesize that the input to the cerebellum, from precerebellar nuclei, could be affected, and thus contributing to the observed reduction of the cerebellar size. During hindbrain development, precerebellar neurons emerge from the proliferative neuroepithelium of the rhombic lip and migrate tangentially to reach their final destinations in the pons and medulla. These neurons form five distinct nuclei that are located at different positions within the hindbrain: pontine gray nuclei (PGN), reticulotegmental nuclei (RTN), external cuneate nuclei (ECN), lateral reticular nuclei (LRN), and inferior olivary nuclei (IO) [41–44]. The PGN, RTN, ECN, LRN projects mossy fibers to the cerebellum, whereas IO projects climbing fibers, and thereby provide input to the cerebellum.[41–44] To tackle the hypothesis that deficits in development of precerebellar nuclei result in abnormalities in cerebellar size of *Arhgef2* mutant mice, we immunostained sagittal and coronal sections taken from *Arhgef2* homozygous and *Arhgef2* heterozygous (used as control) mice with antibodies against the transcription factor Mbh2 (which labels PGN, RTN, ECN, LRN) and FoxP2 (that marks IO). This analysis revealed that *Arhgef2* mutant mice completely lack, in addition to PGN, the RTN and have a severe reduction in LRN neurons (Figs 7A, 7B, 7D, 8A, 8B and 8D). Interestingly, the rostral part of the ECN was abnormally enlarged in *Arhgef2* mutant mice when compared to controls, suggesting that some PGN, RTN and LRN might fail to migrate to their ventral positions and aberrantly locate in the ECN (Figs 7C, 8C and 8D). The formation of the IO was not obviously disturbed in *Arhgef2* mutant mice (Fig 7D). Taken together, our anatomical analysis revealed that specific precerebellar nuclei are either absent or severely reduced in *Arhgef2* mutant mice.

These findings draw our attention to two possible interpretations on the role of Arhgef2 in development of precerebellar nuclei: (1) Arhgef2 modulates the generation of precerebellar neurons and/or (2) Arhgef2 regulates the migration of these cells from their progenitor niche towards their final localization. Precerebellar neurons arise from discrete progenitor domains located in the developing dorsal hindbrain, which are defined by the combinatorial expression of the transcription factor Olig3 with other bHLH (basic Helix-Loop-Helix) transcription factors, such as Atoh1 (Protein atonal homolog 1) or Ptf1a (Pancreas transcription factor 1 alpha).[45] Precerebellar neurons that form the PGN, RTN, LRN and ECN are generated from Olig3/Atoh1 positive (known as dA1) progenitors, whereas those that form the IO emerge from Olig3/Ptf1a positive (known as dA4) progenitors,[46, 47] reviewed in [45]. Arhgef2 is unlikely to modulate generation of precerebellar neurons as its transcript becomes expressed only by E11 in mice, a time point when these cells have already been specified and have started their migration. We thus speculated that Arhgef2 rather modulates precerebellar neuron migration. To test this possibility, we co-stained transverse hindbrain sections, taken from wildtype mice at E11, with an *Arhgef2 in situ* hybridization probe and antibodies against Olig3 and Mbh2 (BarH-like homeobox 1). This revealed that *Arhgef2* is co-expressed largely with Mbh2-positive cells emanating from dA1 progenitors and throughout the anterior and posterior extramural streams precerebellar neurons in the mantle zone of hindbrain (S9 Fig). *Arhgef2* does not follow the expression pattern of Olig3 in dorsal hindbrain (S9 Fig). It is interesting to note that mutation of *Arhgef2* appears to selectively affect the migration of dA1-derived neurons, as other hindbrain centers such as the nucleus of the solitary tract (a Olig3+/Ascl1+ dA3 derivative) or the IO (dA4 derivative) were not affected (Figs 7E and 8E).

This report demonstrates that intellectual disability, mild microcephaly, and a midbrain-hindbrain defect can be caused by a homozygous mutation of the *ARHGEF2* in humans. We highlight the importance of *ARHGEF2* during brain development and, in particular, in neuronal progenitor cell division and differentiation, as well as in neuronal migration. We show that in both human and murine cells *ARHGEF2* deficiency interferes with the normal orientation of mitotic spindles and cell fate choices. As expected, the frameshift mutation of *ARHGEF2* deregulates the activity of its downstream effectors, i.e. the RhoA/ROCK/MLC pathway. ARHGEF2 thereby joins other members of the same protein family that have already been associated with neurologic disease such as non-syndromic intellectual disability (*ARHGEF6*),[30] epileptic encephalopathy (*ARHGEF9*),[31] and peripheral demyelinating neuropathy (*ARHGEF10*).[32] We show that *Arhgef2* deficiency in mice severely impairs the migration of dA1 progenitors, which results in the incorrect location of precerebellar neurons in the hindbrain of *Arhgef2* mutant mice. In keeping with our data, previous studies have shown that deficits in the RhoA/ROCK pathway affect migration of precerebellar neurons.[48] Thus, increasing evidence support the notion that the ARHGEF2-RhoA/ROCK pathway is essential for the migration and specification of precerebellar neurons. ARHGEF2 has been shown to be involved in Wnt-mediated planar cell polarity pathway through its interaction with the Daam and Dishevelled proteins, which are known to control migratory events. *Wnt1* mutant mice have been reported to have a midbrain-hindbrain malformation,[49] and humans with biallelic *WNT1* mutations displayed cerebellum and brainstem phenotype.[50] It is interesting to note that the major effects in the hindbrain of *Arhgef2* mutant mice occur in the migration of dA1-derived neurons, but not other neuronal cell types that emerge from neighboring progenitor domains. Our data demonstrate thus a marked specificity in the molecular control of neuronal migration in the hindbrain. The combined midbrain-hindbrain malformation phenotype observed in humans was not fully recapitulated in *Arhgef2* deficient mice, which lacked an obvious midbrain phenotype. This suggests that during evolutionary divergence of these species, additional molecular mechanisms coordinate the function of ARHGEF2. Identifying additional families with *ARHGEF2* mutations will help to consolidate the disease-causative role of ARHGEF2 in humans.

## Materials and methods

### Ethics statement

The human study was approved by the local ethics committees of the Charité (approval no. EA1/212/08), and the written consent form was received from the investigated individuals. All animal experiments were carried out in accordance to the guidelines of national ethic principles, and approved by Charité (registration no. T0344/12).

### Genetic analyses

DNA samples of the two affected individuals were subjected to whole exome sequencing. Five μg genomic DNA were enriched with the Agilent Human All Exon V3 kit (Agilent, Santa Clara, CA, USA) following the manufacturer’s protocol. The libraries were sequenced using Illumina HiSeq 2000 sequencer for single-end 101 bp. Coverage of coding regions was >92.6–93.4% with a minimal depth of 20-fold; the raw data were processed as described previously.[51] In brief, the raw sequences were aligned to human reference genome (hg19), and the alignment results were used to call variants of SNVs, indels, and CNVs. We defined from the exome data the regions of homozygosity by at least four high-quality (genotype quality ≥ 20, allele counts ≥ 10, and allele percentage ≥ 0.9) consecutive homozygous SNVs uninterrupted by heterozygous SNVs. The overlapping regions of homozygosity (>1 Mb) between the two patients were listed in S4 Table. Subsequently, the variants were screened by known causal mutation databases (OMIM and HGMD), and polymorphism databases (1000Genome, ESP6500, dbSNP138, and an in-house exome database with 721 individuals of whom >90% are of the Middle East origin) with matching variants not exceeding the prevalence cutoff of 0.5%, and the pathogenicity of variants was further evaluated (analysis pipeline: https://sourceforge.net/projects/merap/). Sanger sequencing of the *ARHGEF2* (NM_004723.3) in our cases was performed to confirm the mutation in the patients, establish the genotype in the other family members, and evaluate the presence of potential mutations in additional six consanguineous pedigrees of Sri Lankan, Italian and Turkish descent with developmental delay and a similar radiological phenotype of pontine and cerebellar hypoplasia. Some of these patients also had facial dysmorphism, ataxia, autism, epilepsy, and further MRI findings including a migration or myelination defect. The healthy status of heterozygous parents argues against a haploinsufficiency mechanism underlying this disease phenotype. Two patients with heterozygous deletions encompassing the *ARHGEF2* are listed in the Decipher database: (i) patient 276515 (https://decipher.sanger.ac.uk/patient/276515; 2.76 Mb deletion covering 59 genes; arr 1q22;q23(155,296,964–158,056,876)) with abnormal facial features, intellectual disability, and speech delay and (ii) patient 255240 (https://decipher.sanger.ac.uk/patient/255240; 0.92 Mb deletion spanning 24 genes; arr 1q22(155,192,986–156,108,069)) with facial dysmorphism, brachydactyly, intellectual disability, severe speech delay, muscular hypotonia, but normal cMRI findings.

Apart from the *ARHGEF2* candidate variant, there were three further homozygous variants shared by the two patients that we ranked as unlikely disease-causing: (i) *EXTL1* (NM_004455, exostosin-like glycosyltransferase 1), g.1:26356156G>A, c.939G>A, p.W313X. However, there were 16 heterozygotes in the ESP6500 database and one healthy individual in our in-house database with a homozygous variant. (ii) *HMCN1* (NM_031935, hemicentin 1), g.1:186010193G>A, c.6229G>A, p.D2077N. However, this gene has been reported to be associated with macular degeneration, a phenotype that does not fit the clinical features of our index patients. (iii) *IGFN1* (NM_001164586, immunoglobulin-like and fibronectin type III domain containing 1), g.1:201193885G>A, c.10369G>A, p.G3457S. However, there were 7 heterozygotes in the ESP6500 database, and this variant has a prediction score from SIFT (tolerated) and PolyPhen2 (possibly damaging) that makes it unlikely as well as a GERP conservation score of merely 1.101.

Because both patients were male, we checked on the X chromosome for hemizygous variant, but detected no rare deleterious variant shared by the two individuals.

### Epstein-Barr virus-transformed LCLs

LCLs were established and cultured according to the protocol published by Neitzel et al. 1986.[52]

### Quantitative real-time PCR (qPCR)

RNA extraction and cDNA synthesis were performed with established methods reported previously.[53] To specifically amplify and detect *ARHGEF2* and *RPII* (RNA polymerase II, reference gene) cDNA, we designed sets of primers using the Primer3 online software (www.primer3.ut.ee). qPCR experiments were run in triplicate using Maxima SYBR Green/ROX qPCR Master Mix (Thermo Scientific, Braunschweig, Germany) according to the manufacturer’s protocol with primers specified in S5 Table. Quantification was performed as described previously,[53] and statistical calculations were performed on GraphPad Prism 5 Software (GraphPad Software Inc., La Jolla, CA, USA).

### Western blot

Protein extraction and Western blots (run in triplicates) were performed with established methods reported previously;[54] antibodies are listed in S6 Table.

### Immunocytology

LCLs briefly plated on poly-L-lysine (Sigma-Aldrich, Taufkirchen, Germany) -coated coverslips were fixed in 4% PFA. Coverslips were further incubated in staining buffer (0.2% gelatin, 0.25% Triton X-100, 10% donkey normal serum) for 30 min for permeabilization and blocking, followed by overnight incubation with primary antibodies and an 2 h incubation with the corresponding secondary antibodies (antibodies listed in S6 Table). Nuclei were labeled with 4’,6-Diamidino-2-phenylindole (DAPI, 1:1000, Sigma-Aldrich). Fluorescently labeled cells were analyzed and imaged using a fluorescent Olympus BX51 microscope with the software Magnafire 2.1B (2001) (Olympus, Hamburg, Germany), and images were processed using Adobe Photoshop and ImageJ.

### Cell cycle analysis

Peripheral blood lymphocytes (PBL) were isolated from heparinized blood samples by Ficoll density gradient centrifugation. DNA damage was introduced by exposure of PBLs to 6 MV X-ray photons. Matched cultures were set up from untreated and irradiated cells in RPMI 1640 medium, supplemented with 15% fetal bovine serum (FBS, PAN Biotech, Aidenbach, Germany). Lymphocyte growth activation was achieved by phytohemagglutinin (PHA HA16, Thermo Scientific, Dartford, UK). The cell cycle assay was performed using 5-bromo-2′-deoxyuridine (BrdU)–Hoechst 33258 flow cytometry. Cells with replication fork-stalling types of DNA damage become delayed in the G2 phase of the cell cycle.

### Phospho-histone H3 analysis

LCLs were washed in PBS, fixed in 2% paraformaldehyde (PFA), and permeabilized with 90% methanol for 30 min on ice. Phospho-histone H3-Ser10 (pH3) was detected with anti-pH3 antibody and corresponding secondary antibody (S6 Table). DAPI at 2 μg/ml final concentration was used as a counterstain for DNA content and cell cycle distribution. Fluorescence was recorded using the same LSRII flow cytometer (Becton Dickinson, Franklin Lakes, NJ, USA) as for cell cycle studies. Data analysis was done with WinMDI 2.9 software (MicroSoft, Redmond, WA, USA).

### Cortical precursor cultures

Cortices of *CD1* mouse E13 embryos (Charles River, Sherbrooke, Quebec, Canada; E, embryonic days) were dissected in ice-cold Hank’s balanced salt solution (HBSS, Gibco, Carlsbad, CA, USA) and immediately placed in cortical precursor culture media (CPCM), Neurobasal (Gibco) supplemented by 40 ng/ml bFGF (BD Biosciences, San Jose, CA, USA), 2% B27 (Gibco), 1% Penicillin/Streptomycin (Lonza, Basel, Switzerland), and 500 μM L-Glutamine (Gibco). Following mechanical tissue dissociation, cells were plated on Poly-D-Lysine (Sigma, St.Louis, MO, USA) and mouse laminin (Corning, Corning, NY, USA) -coated coverslips (150,000 viable cells per well of a 24-well plate) and allowed to adhere for four hours in CPCM. Subsequently, cells were transfected with the appropriate plasmids overnight using Lipofectamine LTX (Invitrogen, Carlsbad, CA, USA) in Opti-MEM (Gibco). Because, *Lfc* overexpression is known to cause cell death, following transfection, 50 μM of the caspase inhibitor ZVAD-FMK (R&D Systems, Minneapolis, MN, USA) was added to the culture media.[24] Two days later, on DIV3, cells were fixed with 4% PFA and permeabilized with 0.2% NP-40 (USB Corporation, Cleveland, OH, USA) in PBS. Cultures were then stained with antibodies listed in S6 Table. EGFP-positive cells (n = 75–100 per cover slip, prepared in four independent experiments) were evaluated for co-staining with βIII-tubulin or Ki67 using a Zeiss Axiovert A.1 fluorescence microscope.

### Plasmids

The nuclear EGFP (*pEF*-*EGFP*) expression plasmid and the *Arhgef2 (Lfc)* shRNA constructs in *pG-SHIN2* vector were generated as reported previously.[24] Wildtype *ARHGEF2* (NM_001162383.1) and mutant *ARHGEF2* (c.1462delG, position according to NM_004723.3) were cloned into a *pcDNA3*.*1(Zeo)(+)* plasmid (Invitrogen). Mutant *ARHGEF2* was generated using the QuikChange II XL Site-Directed Mutagenesis kit (Agilent, 200521) with primers given in S5 Table following the manufacturer’s protocol. Sanger sequencing confirmed the correct insertion of the mutation.

### *In utero* electroporation

*In utero* electroporation was performed as described previously[55] using a CUY21 EDIT electroporator (TR Tech, Japan) to deliver five 50 ms pulses of 40–50 V with 950 ms intervals per embryo. Per embryonic brain, 4 μg total of plasmid DNA was suspended in the tracer 0.5% trypan blue in a ratio of 37.5% *shArhgef2 (Lfc)* (or shCon), 37.5% human *ARHGEF2* (wt)/human *ARHGEF2* (mut)/empty vector, and 25% *pEF-EGFP* plasmid. Embryos were collected 3 days following electroporation. Following an over-night fixation in 4% PFA, brains were cryopreserved and mounted. Coronal cryosections of 18 μm were incubated with antibodies listed in S6 Table. Images were collected from three brain sections per condition using a Quorom spinning disk confocal microscope system or the Zeiss Axio Imager M2 system. EGFP-positive cells were counted in either the combined ventricular zone/sub-ventricular zone/intermediate zone (VZ/SVZ/IZ) or the cortical plate (CP) and reported as proportion of EGFP-positive cells in each of these regions. SatB2/EGFP-double positive cells in the CP were also counted for each condition.

For mitotic spindle plane analysis, the electroporated brain sections were stained with antibodies directed against Cdk5rap2 and corresponding secondary antibodies (S6 Table). Nuclei were labeled with DAPI. Images of transfected cells in 10–15 μm z-stacks at an interval of 0.1 μm thickness were collected using a Zeiss Spinning Disc microscopy system CXU-S1 with ZEN 2012 software. The mitotic spindle plane was evaluated through measurement of the angle between the ventricular surface and a line connecting the centrosomes, using the ImageJ software.

### RhoA Pull-down assay

Activated RhoA was assessed using the Rho Activation Assay Biochem kit (Cytoskeleton, Denver, USA) according to the manufacturer’s protocol. Experiments were run in triplicate.

### Analysis of *Arhgef2* deficient mice

*Arhgef2* deficient mice were genotyped as previously reported.[40] P0 and adult brains were dissected and embedded in paraffin/OCT medium as reported previously. [54] Sagittal/coronal brain sections of 10 μm were collected on histologic slides and stained with Hematoxylin and Eosin (H&E) or Gallyas or 3, 3'-Diaminobenzidine (DAB) staining following standard protocols. Whole brain, brain stem, cerebellum, and pontine nuclei volume were evaluated by measuring respective areas in every tenth section using ImageJ and multiplying by the thickness between sections. Immunohistology was performed on brain sections by blocking in staining buffer (0.2% gelatin, 0.25% Triton X-100, 3% BSA) for 30 min for permeabilization, followed by overnight incubation with primary antibodies and an 2 h incubation with the corresponding secondary antibodies (antibodies listed in S6 Table). Nuclei were labeled with 4’,6-Diamidino-2-phenylindole (DAPI, 1:1000, Sigma-Aldrich). Images were obtained using a Zeiss Spinning Disc microscopy system CXU-S1 with ZEN 2012 software.

### *In situ* hybridization of *Arhgef2*

A 516 bp *Arhgef2* PCR product was generated with primers listed in S5 Table and subsequently cloned into a p-AL2-T vector. The RNA probe was generated by *in vitro* transcription. Chromogenic/fluorescence *in situ* hybridization was performed on 16 μm thick brain sections from E11/P0 mice post-fixed in 4% PFA for 15 min and permeabilized with Proteinase K for 2.5 min at RT. The reaction was stopped by applying 0.2% glycine in PBS 1x, followed by 20 min post fixation with 20% glutaraldehyde in 4% PFA. The sections were treated with the pre-hybridization buffer (50% formamide, 5x SSC pH 7.0, 2.5 M EDTA, 0.1% Tween-20, 0.15% CHAPS, 0.1 mg/ml Heparin, 100 μg/ml yeast tRNA, 50 μg/ml salmon sperm DNA, 1x Denhardt’s solution) at 65°C for 2 hours followed by overnight incubation with *Arhgef2* RNA probes at 65°C. Sections were then treated with RNase A for 30 min at 37°C, subsequently washed with SSC pH 4.5 in 50% formamide at 65°C and then with KTBT (100 mM NaCl, 50 mM Tris–HCl pH 7.5, 10 mM KCl, 1% Triton X-100) at RT. Following blocking in 20% sheep serum and incubation with sheep anti-DIG antibody (1:1,000, Roche, Mannheim, Germany) at 4°C overnight, the sections were washed in KTBT and NTMT (50 mM NaCl, 100 mMTris–HCl pH 9.5, 50 mM MgCl_2,_ 0.5% Tween-20) for 1 hour at RT. Development was achieved through addition of the chromogenic NBT/BCIP substrate (1:50, Roche).

### Proliferation and cell size assay of WT and *Arhgef2*-/- mice

CD4^+^ naive T cells were isolated from spleen of *C57BL*/6 (WT) and *Arhgef2*-/- mice by using CD4^+^CD62L^+^ T Cell Isolation Kit (Miltenyi Biotec, San Diego, CA, USA) and subsequently stained with CellTracer Violet Cell Proliferation Kit (Thermo Fisher Scientific) according to the instructions provided. Cells were then incubated with 2 μg/ml of anti-CD28 antibody (BD, New Jersey, USA) in anti-CD3e antibody (BD, New Jersey, USA) pre-coated wells for 4 days at the cell concentration of 1 × 10^6^ cell/ml. 4 days later, cells were collected and stained with APC-conjugated anti-CD3e (Biolegend, San Diego, CA, USA) and BV786-conjugated anti-CD4 (BD, New Jersey, USA). Cells were analyzed by FACS Aria II (BD Bioscience, New Jersey, USA) followed by using FlowJo software (Tree Star). All cells were pre-gated on singlet cells (area & width) and on living cells (7AAD^−^).

## Supporting information

S1 Table Phenotype of patients with homozygous *ARHGEF2* mutation. (PDF)

S2 Table Clinical growth chart from both affected brothers. (PDF)

S3 Table Development of affected patients from two months through two years. (PDF)

S4 Table Regions of homozygosity (> 1 Mb) between patients II.1 and II.2. (PDF)

S5 Table Primer sequences for qPCR, *in situ* hybridization, and site-directed mutagenesis. (PDF)

S6 Table List of primary antibodies. (PDF)

S1 Fig Full-length Western blot of ARHGEF2. ARHGEF2 can be detected at the expected height of 120 kDa in control LCLs and LCLs of heterozygous parents. Both full length (120 kDa) and the predicted truncated (57 kDa) ARHGEF2 could not be detected in patient LCLs using N-terminus antibody in Western blot analysis. Actin (43 kDa) is used as loading control.
(TIF)

S2 Fig Normal mitotic transit and minimal sensitivity of P1 (II.1) and P2 (II.2) patient cells towards ionizing radiation. (**A**) Normal proportions of H3P (Ser10)-positive cells (y-axis, boxed) and uni-parametric cell cycle distributions (x-axis) in LCL culture of patients (II.1, II.2), their parents and a normal control. **(B) (i)** Cell cycle allocation in a 72-h lymphocyte culture from patient P2 without prior irradiation. Bivariate BrdU–Hoechst 33258 and PI flow cytometry shows the distribution of cells within up to four cell cycles, I (G0/G1 to G2), II (G1′ to G2′), III (G1″ to G2″), and IV (G1‴). (**ii**) Exposure of lymphocytes to 1.5 Gy irradiation at culture setup results in a more pronounced track of debris from the G0/G1 phase, slight growth reduction and minimal accumulation of cells in G2, similar to what is observed in normal controls. (**iii**) A standard dosage level of 1.5 Gy discriminates normal control (CON, gray circles; n = 75; mean ± 1 SD, 0.09±0.03) from AT lymphocyte cultures (gray diamonds; n = 77; mean ± 1 SD, 0.41 ± 0.13). P1 (black upright triangle; G2 ÷ GF, 0.14) and P2 (black inverted triangle; G2 ÷ GF, 0.10) lymphocytes fall within the range of normal control G2 ÷ GF ratios. Other radiosensitive controls include LIG4 (gray squares; mean ± 1 SD, 0.44 ± 0.09) and NHEJ1 (gray hexagons; mean ± 1 SD, 0.63 ± 0.05) lymphocytes. (i**v**) The G2 ÷ GF rates of 72-hr lymphocyte cultures from P1 (black upright triangle) and P2 (black inverted triangle) resemble the dose–response curve seen in normal controls (CON, gray circles; n = 75; means ± 1 SD) rather than that of AT (gray diamonds; n = 11–77; means ± 1 SD), LIG4 (gray squares; n = 1–2; single values or range) or NHEJ1 (gray hexagons; n = 1–3; single values or means ± 1 SD) radiosensitive controls for a broader range of irradiation (0–8 Gy).
(TIF)

S3 Fig Normal centrosomal morphology in the index patients. Representative fluorescence micrographs of control and patient (II.1, II.2) LCLs stained for centrosomal γ-tubulin (green) and DAPI (blue), indicating normal centrosomal integrity. Scale bar 10 μm.
(TIF)

S4 Fig Proliferation rate and T-cell size is not altered in the *Arhgef2-/-* mice. CellTracer Violet stained splenic CD4^+^ naive T cells were incubated under the anti-CD3e / CD28 antibody stimulation for 4 days. Cells were collected and analyzed by flow cytometer. (**A**) Each generation (G0 to G6) were gated as shown, following gated for CD3e^+^ and CD4^+^. (**B**) Bar graph shows proliferation index (PI), using following formula; PI = (N_G0_ + N_G1_ + N_G2_ + N_Gn_) / (N_G0_/2^0^ + N_G1_/2^1^ + N_G2_/2^2^ + N_Gn_/2^n^), n = 6, Error bars indicate mean ± S.E.M, ns = not significant. (**C**) Histograms show FSC and SSC of total CD3e^+^ CD4^+^ T cells (upper) or 4th generation (G4) in CD3e^+^ CD4^+^ T cells (lower).
(TIF)

S5 Fig Expression of *Arhgef2* in E11 and P0 mouse brain. **(A)** *Arhgef2* is strongly expressed in the neuroepithelium of the brain at E11. **(B)** At P0, *Arhgef2* is expressed predominantly in the VZ/SVZ and upper layer of the neocortex, in the external granule layer (EGL) of the cerebellum and in the pontine nuclei (DIC images of *in situ* hybridization with anti-sense and sense *Arhgef2* RNA probes, n = 4, scale bar 1 mm (A), scale bar 100 μm (B)).
(TIF)

S6 Fig *Arhgef2-/-* mice shows normal distribution of neurons in the cortical layers. **(A)** Representative fluorescence micrographs of WT and *Arhgef2-/-* mice cortex stained for layers II-IV (Cux1, green), layers V-VI (Ctip2, red) and DAPI. **(B)**. Quantification of cortical volume, surface area, thickness, total number of DAPI cells, Cux1 and Ctip2 positive cells per view field revealed no significant difference between control and mutant mice. (n = 3–4, Student’s *t-test*, ns- not significant, error bars represent ± SD, scale bar 100 μm).
(TIF)

S7 Fig *Arhgef2-/-* mice shows no abnormalities in the midbrain structures. Representative DAB (3, 3'-Diaminobenzidine) stained micrographs of WT and *Arhgef2-/-* mice showing normal structures of FoxP2-positive inferior colliculus and superior colliculus. Scale bar 200 μm.
(TIF)

S8 Fig Normal Purkinje cell size and molecular layer thickness in the *Arhgef2-/-* mice. **(A)** Representative H&E stained micrographs of WT and mutant cerebellum (scale bar 500 μm), and **(B)** magnified cerebellar folia. **(C)** No significant change in Purkinje cell size and thickness of the molecular layer was observed in the mutant mice upon quantification. (n = 3–4, Student’s *t-test*, ns- not significant, error bars represent ± SD, scale bar 20 μm).
(TIF)

S9 Fig Co-expression of *Arhgef2* and Mbh2 in the mantle zone of developing hindbrain. Photomicrographs representing transverse E11 section of dorsal hindbrain stained for *Arhgef2* (green), Mbh2 (red), Olig3 (cyan), and DAPI, indicates a clear colocalisation in the mantle zone along migratory pathway. Scale bar 100 μm.
(TIF)
