# Supplementary material for: Homozygous ARHGEF2 mutation causes intellectual disability and midbrain-hindbrain malformation
Source: PLoS Genet. 2017 Apr 28;13(4):e1006746. doi: 10.1371/journal.pgen.1006746 (PMC5428974; doi:10.1371/journal.pgen.1006746)
Supplement: S1 Table — (PDF) [file pgen.1006746.s001.pdf]

S1 Table. Phenotype of patients with homozygous *ARHGEF2* mutation.

**Characteristics and Symptoms**	**HPO ID[a]**	**Patient**	**Patient**
Pedigree ID	NA	II.1	II.2
Origin	NA	Kurd	Kurd
Gender	NA	male	male
Age at last assessment (years)	NA	5.9	4.5
**Head**			
(Infantile) congenital microcephaly (OFC < P3)	0011451	+	(+)
High palate	0000218	+	-
Downslanted palpebral fissures	0000494	+	-
Long eyelashes	0000527	+	+
Bilateral ptosis	0001488	+	-
Congenital strabismus	0000487	+	+
Astigmatism	0000483	+	+
Horizontal pendular nystagmus	0007811	-	+
Amblyopia	0000646	-	+
Optic disc pallor	0000543	-	+
Abnormality of the retinal pigmentation	0008051	-	+
**Chest**			
Wide intermamillary distance	0006610	+	-
**Skeletal**			
Broad finger	0001500	+	-
**Skin, Hair**			
Skin rash	0000988	+	-
Low posterior hairline	0002162	+	-
**Neurologic**			
Intellectual disability, mild (IQ equivalent; years at assessment)	0001256	+ (68; 1.5)	-
Intellectual disability, moderate (IQ equivalent; years at assessment)	0002342	+ (<50; 2.2)	+ (46; 0.6)
Delayed speech and language development	0000750	+	+
Muscular hypotonia	0001252	+	-
Hypoplasia of the pons	0012110	+	+
Cerebellar vermis hypoplasia	0001320	-	+
Abnormal auditory evoked potentials	0006958	+	-
Abnormality of vision evoked potentials	0000649	-	+

[a]All symptoms are listed according to the nomenclature and the systematics of the OMIM "Clinical Synopsis" and the Human Phenotype Ontology (HPO; http://www.human-phenotype-ontology.org/) according to Robinson et al. 2008.(1) Abbreviations: AR, autosomal recessive; HPO, human phenotype ontology; IQ, intelligence quotient; OFC, occipito-frontal head circumference; NA, not applicable; OMIM, (Online Mendelian Inheritance in Man); P, percentile.

References

1. Robinson PN, Kohler S, Bauer S, Seelow D, Horn D, Mundlos S. The Human Phenotype Ontology: a tool for annotating and analyzing human hereditary disease. Am J Hum Genet. 2008;83(5):610-5.
