# Supplementary material for: Homozygous ARHGEF2 mutation causes intellectual disability and midbrain-hindbrain malformation
Source: PLoS Genet. 2017 Apr 28;13(4):e1006746. doi: 10.1371/journal.pgen.1006746 (PMC5428974; doi:10.1371/journal.pgen.1006746)
Supplement: S2 Table — (PDF) [file pgen.1006746.s002.pdf]

S2 Table. Clinical growth chart from both affected brothers.

Age (years)	OFC (cm)	Centile	SDS	Height (cm)	Centile	SDS	Weight (kg)	Cen-tile	SDS	BMI (kg/m²)	Centile	SDS
**Patient (pedigree ID II.1)**												
0.0 (40 GW)	34.5	2.6	-1.95	53.0	85	1.04	3.48	75	0.13	12.5	23	-0.72
0.96	43.0	0.1	-3.04	77.0	72	0.59	9.3	20	-0.03	15.7	5	-1.61
1.25	ND	-	-	80.0	58	0.20	9.9	40	-0.59	15.5	6	-1.58
1.60	ND	-	-	85.0	65	0.38	11.3	40	-0.05	15.6	13	-1.11
2.01	ND	-	-	87.5	44	-0.15	11.4	20	-1.06	14.9	7	-1.48
2.10	46	0.4	-2.64	ND	-	-	11.6	10	-0.93	ND	-	-
2.32	ND	-	-	90.0	40	-0.25	12.5	40	-0.39	15.4	20	-0.83
2.52	ND	-	-	90.0	23	-0.72	12.5	10	-0.95	15.4	22	-0.76
3.30	48.0	1.4	-2.20	97.0	27	-0.61	14.0	30	-0.45	14.9	16	-0.99
5.94	48.5	0.3	-2.79	113	21	-0.82	15.8	<3	-1.53	12.4	<0.1	-3.29
**Patient (pedigree ID II.2)**												
0.0 (39 GW)	34	1.0	-2.33	52	72	0.59	3.4	70	0.02	12.6	27	-0.62
2.25	47	2.0	-2.05	88	28	-0.59	12.8	40	-0.21	16.5	51	0.03
4.51	49.5	5	-1.61	105	24	-0.71	17.5	45	-0.25	15.9	58	0.20

Centiles and SDS of height according to Kronemeyer-Hauschild K et al 2001,(1) of weight according to WHO Growth Charts (WHO Multicentre Growth Reference Study, MGRS, 1997-2003, www.who.int/childgrowth/standards/), and of OFC according to Prader A et al 1989.(2) Abbreviations: BMI, body mass index; GW, gestational week; ND, not determined; OFC, occipital-frontal head circumference; SDS, standard deviation score.

References

1. Kromeyer-Hauschild K WM, Kunze D, Geller F, Geiß HC, Hesse V, von Hippel A, Jaeger U, Johnsen D, Korte W, Menner K, Müller G, Müller JM, Niemann-Pilatus A, Remer T, Schaefer F, Wittchen H-U, Zabransky S, Zellner K, Ziegler A, Hebebrand J. Perzentile für den Body-mass-Index für das Kindes- und Jugendalter unter Heranziehung verschiedener deutscher Stichproben. . Monatsschrift Kinderheilkunde 2001;149:807-18.
2. Prader A, Largo RH, Molinari L, Issler C. Physical growth of Swiss children from birth to 20 years of age. First Zurich longitudinal study of growth and development. Helv Paediatr Acta Suppl. 1989;52:1-125.
