# Supplementary material for: Homozygous ARHGEF2 mutation causes intellectual disability and midbrain-hindbrain malformation
Source: PLoS Genet. 2017 Apr 28;13(4):e1006746. doi: 10.1371/journal.pgen.1006746 (PMC5428974; doi:10.1371/journal.pgen.1006746)
Supplement: S3 Table — (PDF) [file pgen.1006746.s003.pdf]

S3 Table. Development of affected patients from two months through two years.

**Characteristics and Features**	**Norms (months)[a]**	**Patient**	**Patient**
Pedigree ID	NA	II.1	II.2
Lifts head up for several seconds while prone	2	9	ND
Rolls from prone to supine	5-6	ND	6
Babbles	5-6	ND	7
Says „da-da“, „ba-ba“	7 - 8	ND	11
Sits well without support	9-10	9	ND
Pulls self to sit	9-10	9.5	ND
Walks with assistance	11-12	ND	11
Uses two to four words with meaning	11-12	39	27
Creeps well	11-12	9	11
Walks by self	13-15	18	15
Speaks in two- or three-word sentences	24	75 (two-word sentences only)	55 (two-word sentences only)

[a]Normal values according to Swaiman KF 2006. [1] Abbreviations: NA, not applicable; ND, not determined.

References

1. Swaiman K. Pediatric Neurology: Principles & Practice: Neurologic examination after the newborn period until 2 years of age. . 1 vol. 4 ed. Philadelphia, USA: Mosby, 2006
