# Supplementary material for: Homozygous ARHGEF2 mutation causes intellectual disability and midbrain-hindbrain malformation
Source: PLoS Genet. 2017 Apr 28;13(4):e1006746. doi: 10.1371/journal.pgen.1006746 (PMC5428974; doi:10.1371/journal.pgen.1006746)
Supplement: S4 Table — (PDF) [file pgen.1006746.s004.pdf]

S4 Table. Regions of homozygosity (> 1 Mb) between patients II.1 and II.2.

chr1:22336277-35184084
chr1:53990321-59248813
chr1:103352451-120512104
chr1:145517561-214504604 (ROH containing *ARHGEF2*)
chr1:240939391-248512498
chr2:56411817-66667186
chr2:143913004-158401137
chr3:71015021-73004157
chr3:105400846-13340249
chr4:33240041-36081878
chr4:167932327-169086441
chr5:38959794-56416688
chr5:99921841-105752888
chr5:172659511-173907351
chr6:20144637-22570064
chr6:97677118-99283376
chr7:118591040-120613419
chr7:155750033-156762446
chr8:42959007-47757290
chr8:126369898-128099269
chr8:139889972-141034189
chr9:9090876-11012433
chr9:80919756-82189914
chr9:122001000-123671520
chr10:1421516-2797555
chr11:25100229-26552702
chr11:83691458-85373668
chr11:130749715-132177633
chr12:29650702-30815157
chr12:90147264-91334194
chr13:67802339-69559248
chr13:81230929-83719952
chr14:86088056-88416390
chr15:95399291-97326748
chr16:58743454-60688861
chr17:53638670-54939715
chr18:68893756-70209321
chr20:18531499-19634747
chr21:21112411-22841123
chr22:29130300-36695247
