# Supplementary material for: Homozygous ARHGEF2 mutation causes intellectual disability and midbrain-hindbrain malformation
Source: PLoS Genet. 2017 Apr 28;13(4):e1006746. doi: 10.1371/journal.pgen.1006746 (PMC5428974; doi:10.1371/journal.pgen.1006746)
Supplement: S5 Table — (PDF) [file pgen.1006746.s005.pdf]

S5 Table. Primer sequences for qPCR, *in situ* hybridization, and site-directed mutagenesis.

**Primer**	**Sequence (5’->3’)**	**Species**
hARHGEF2-F	ACCTTCATCCCCTTCCAGAT	human
hARHGEF2-R	GGCAGGAAAGCAAGAAGAGA	human
hARHGEF2- F	CCCTCATCTGCCCAACCTG	human
hARHGEF2- R	CAAGGCGGTGTTGTTCTTCA	human
hRPII- F	GCACCACGTCCAATGACAT	human
hRPII- R	TCATGAGTGGTCCTGTGTCC	human
hARHGEF2- F	CATCTTTCCTACCCTGACAAGCCTTCAGTGG	human
hARHGEF2- R	CCACTGAAGGCTTGTCAGGGTAGGAAAGATG	human
Arhgef2-F	TCCCTTATTGATGAAGGTGTAGAAG	mouse
Arhgef2-R	GTCTCGAGCATACAGCTCCTTATAG	mouse
