# Supplementary material for: Homozygous ARHGEF2 mutation causes intellectual disability and midbrain-hindbrain malformation
Source: PLoS Genet. 2017 Apr 28;13(4):e1006746. doi: 10.1371/journal.pgen.1006746 (PMC5428974; doi:10.1371/journal.pgen.1006746)
Supplement: S6 Table — (PDF) [file pgen.1006746.s006.pdf]

**S6 Table. List of primary antibodies.**

| Antibody | Source | Catalog No. | Host | Dilution |
|---|---|---|---|---|
| Actin | Millipore | MAB1501 | Mouse monoclonal | 1:10,000 |
| Alpha-tubulin | Sigma-Aldrich | T9026 | Mouse monoclonal | 1:1500 |
| ARHGEF2(55B6) | Cell Signaling | 4076 | Rabbit monoclonal | 1:1000 |
| ARHGEF2 | Cell Signaling | 4145 | Rabbit polyclonal | 1:1000 |
| β-III-tubulin | Covance | MMS-435P-250 | Mouse monoclonal | 1:1000 |
| CDK5RAP2 (N1) | Self-generated | - | Rabbit polyclonal | 1:10,000 |
| CDK5RAP2 | Sigma-Aldrich | HPA035820 | Rabbit polyclonal | 1:200 |
| GFP | Abcam | AB13970 | Chicken polyclonal | 1:1000 |
| γ-tubulin | Sigma-Aldrich | T6557 | Mouse monoclonal | 1:500 |
| Ki67 | BD Pharmingen | 556003 | Mouse monoclonal | 1:200 |
| MLC-2 | Cell Signaling | 3672 | Rabbit polyclonal | 1:1000 |
| Mbh2 | Novus Biologicals | NBP1-86513 | Rabbit polyclonal | 1:200 |
| p-MLC (Ser 19) | Cell Signaling | 3671 | Rabbit polyclonal | 1:1000 |
| RhoA | Cytoskeleton | ARH03 | Mouse monoclonal | 1:500 |
| Phalloidin | Sigma-Aldrich | P1951 | -- | 1:100 |
| Phospho-histone H3 (Ser10) (p-H3) | Cell Signaling | 9706 | Mouse monoclonal | 1:25 |
| Tlx3 | Self-generated | - | Guinea pig polyclonal | 1:10,000 |
| SatB2 | Abcam | ab92446 | Rabbit monoclonal | 1:400 |
| Vinculin | Sigma-Aldrich | V9131 | Mouse monoclonal | 1:10,000 |
| Olig3 | Self-generated | - | Guinea pig polyclonal | 1:10,000 |
