# Supplementary figures and images for: Homozygous ARHGEF2 mutation causes intellectual disability and midbrain-hindbrain malformation
Source: PLoS Genet. 2017 Apr 28;13(4):e1006746. doi: 10.1371/journal.pgen.1006746 (PMC5428974; doi:10.1371/journal.pgen.1006746)

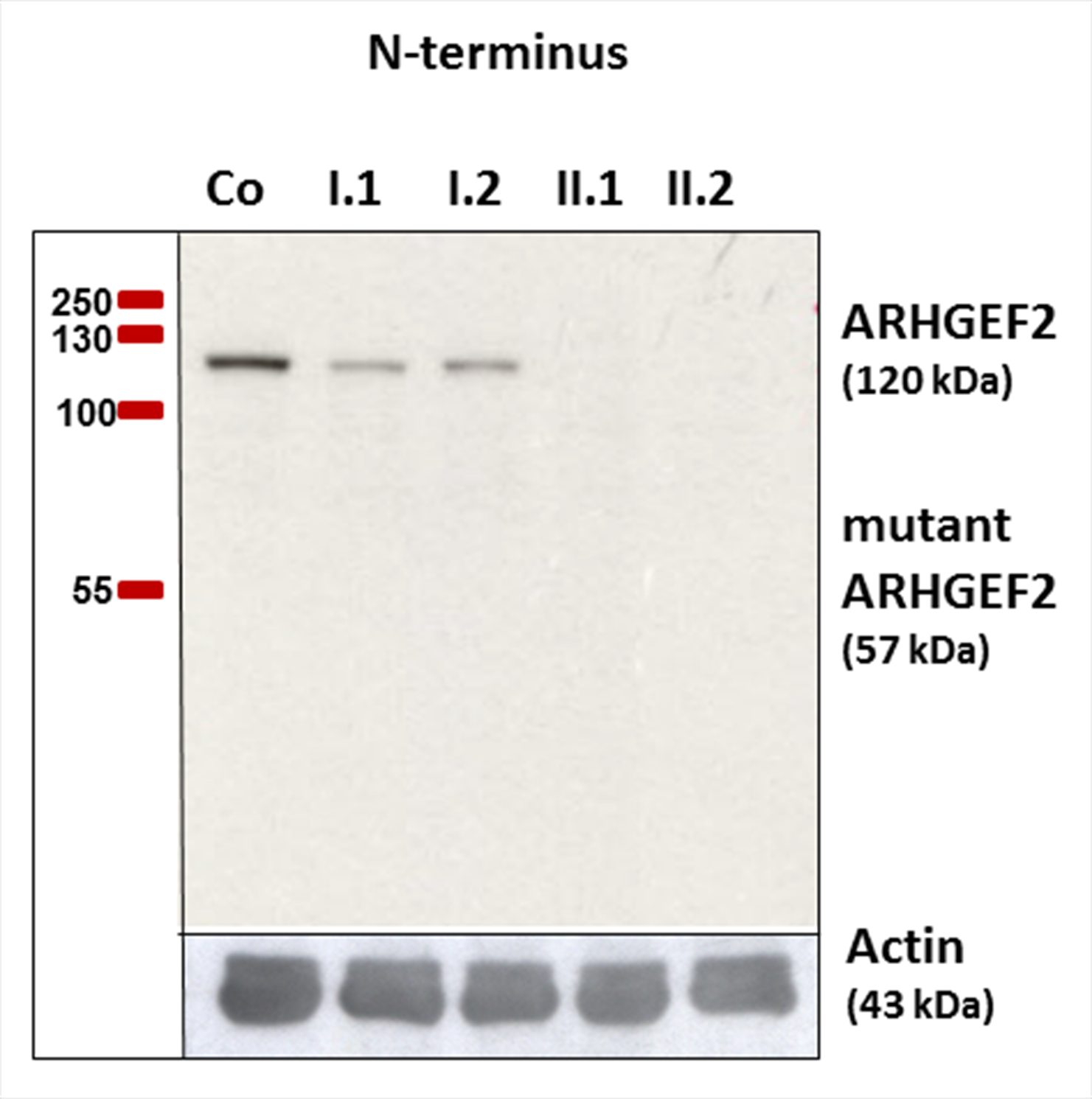

Supplement: S1 Fig — ARHGEF2 can be detected at the expected height of 120 kDa in control LCLs and LCLs of heterozygous parents. Both full length (120 kDa) and the predicted truncated (57 kDa) ARHGEF2 could not be detected in patient LCLs using N-terminus antibody in Western blot analysis. Actin (43 kDa) is used as loading control. (TIF) [file pgen.1006746.s007.tif]

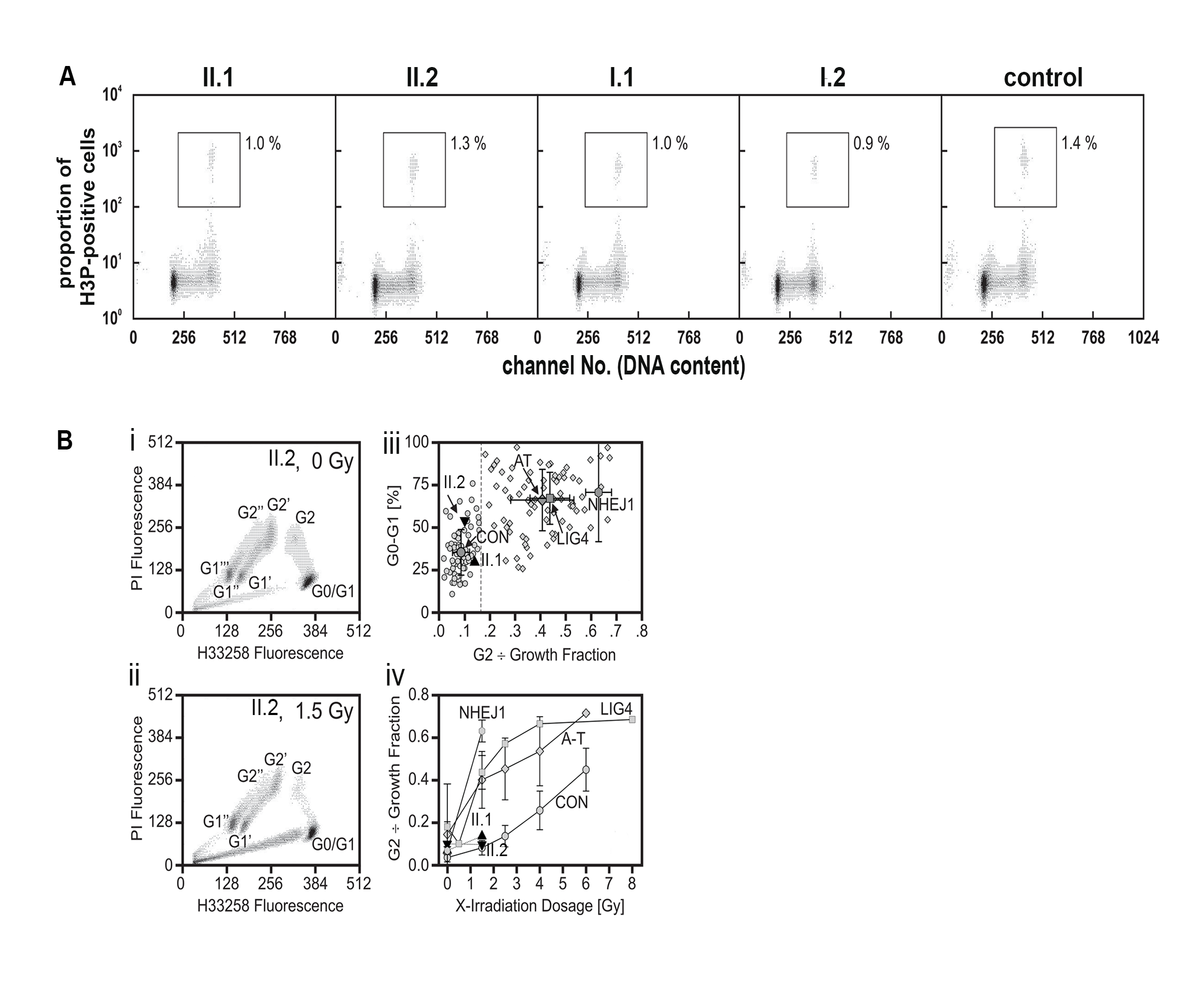

Supplement: S2 Fig — (A) Normal proportions of H3P (Ser10)-positive cells (y-axis, boxed) and uni-parametric cell cycle distributions (x-axis) in LCL culture of patients (II.1, II.2), their parents and a normal control. (B) (i) Cell cycle allocation in a 72-h lymphocyte culture from patient P2 without prior irradiation. Bivariate BrdU–Hoechst 33258 and PI flow cytometry shows the distribution of cells within up to four cell cycles, I (G0/G1 to G2), II (G1′ to G2′), III (G1″ to G2″), and IV (G1‴). (ii) Exposure of lymphocytes to 1.5 Gy irradiation at culture setup results in a more pronounced track of debris from the G0/G1 phase, slight growth reduction and minimal accumulation of cells in G2, similar to what is observed in normal controls. (iii) A standard dosage level of 1.5 Gy discriminates normal control (CON, gray circles; n = 75; mean ± 1 SD, 0.09±0.03) from AT lymphocyte cultures (gray diamonds; n = 77; mean ± 1 SD, 0.41 ± 0.13). P1 (black upright triangle; G2 ÷ GF, 0.14) and P2 (black inverted triangle; G2 ÷ GF, 0.10) lymphocytes fall within the range of normal control G2 ÷ GF ratios. Other radiosensitive controls include LIG4 (gray squares; mean ± 1 SD, 0.44 ± 0.09) and NHEJ1 (gray hexagons; mean ± 1 SD, 0.63 ± 0.05) lymphocytes. (iv) The G2 ÷ GF rates of 72-hr lymphocyte cultures from P1 (black upright triangle) and P2 (black inverted triangle) resemble the dose–response curve seen in normal controls (CON, gray circles; n = 75; means ± 1 SD) rather than that of AT (gray diamonds; n = 11–77; means ± 1 SD), LIG4 (gray squares; n = 1–2; single values or range) or NHEJ1 (gray hexagons; n = 1–3; single values or means ± 1 SD) radiosensitive controls for a broader range of irradiation (0–8 Gy). (TIF) [file pgen.1006746.s008.tif]

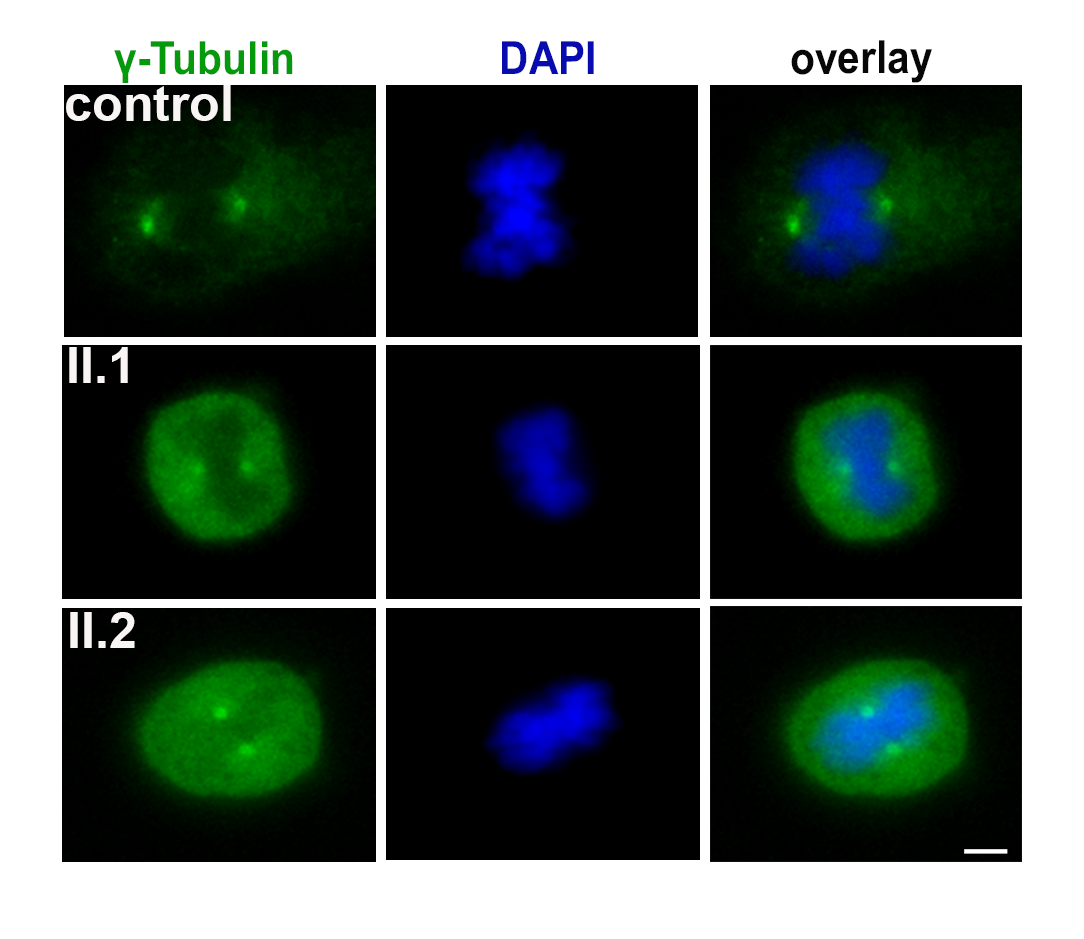

Supplement: S3 Fig — Representative fluorescence micrographs of control and patient (II.1, II.2) LCLs stained for centrosomal γ-tubulin (green) and DAPI (blue), indicating normal centrosomal integrity. Scale bar 10 μm. (TIF) [file pgen.1006746.s009.tif]

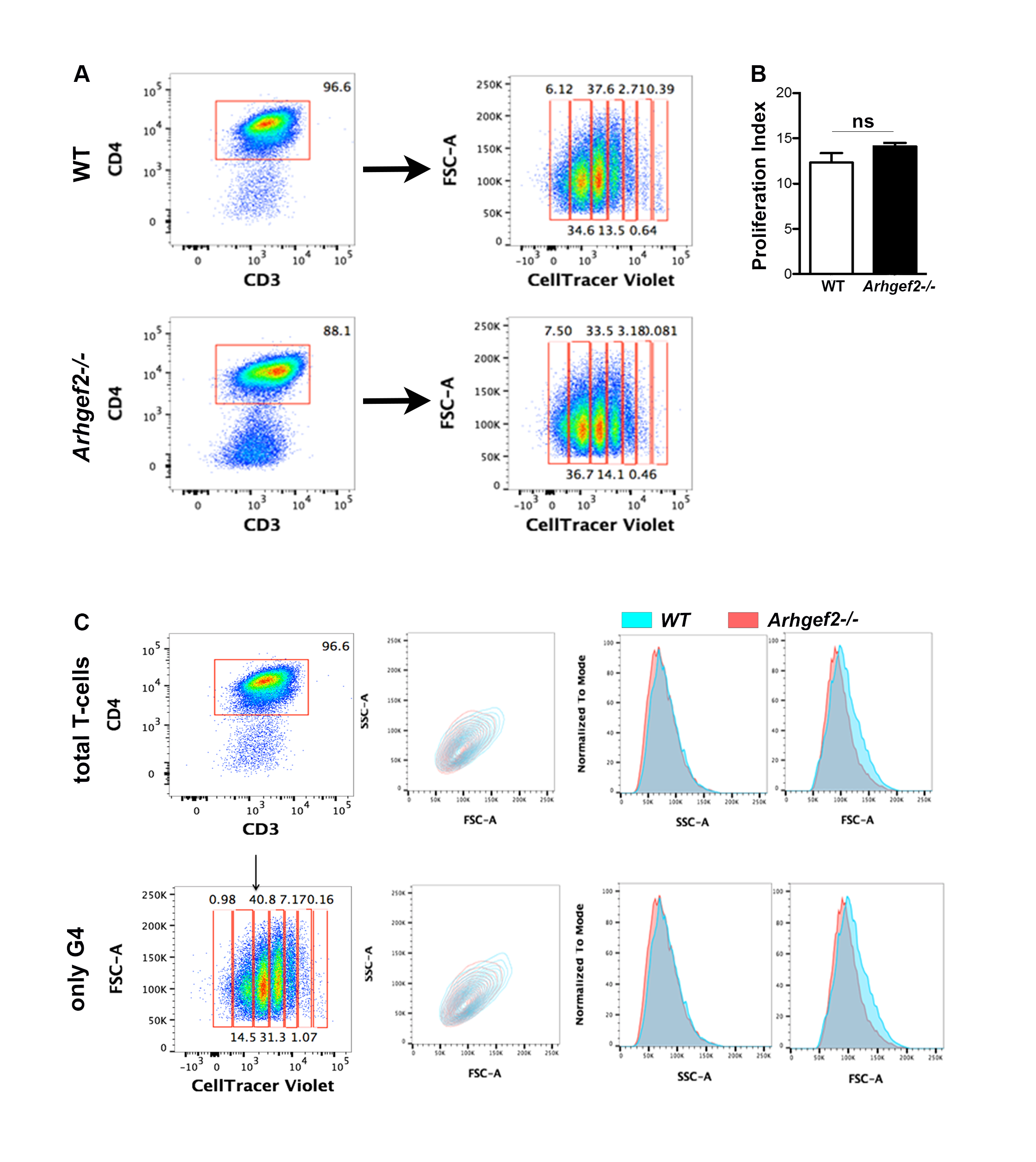

Supplement: S4 Fig — CellTracer Violet stained splenic CD4+ naive T cells were incubated under the anti-CD3e / CD28 antibody stimulation for 4 days. Cells were collected and analyzed by flow cytometer. (A) Each generation (G0 to G6) were gated as shown, following gated for CD3e+ and CD4+. (B) Bar graph shows proliferation index (PI), using following formula; PI = (NG0 + NG1 + NG2 + NGn) / (NG0/20 + NG1/21 + NG2/22 + NGn/2n), n = 6, Error bars indicate mean ± S.E.M, ns = not significant. (C) Histograms show FSC and SSC of total CD3e+ CD4+ T cells (upper) or 4th generation (G4) in CD3e+ CD4+ T cells (lower). (TIF) [file pgen.1006746.s010.tif]

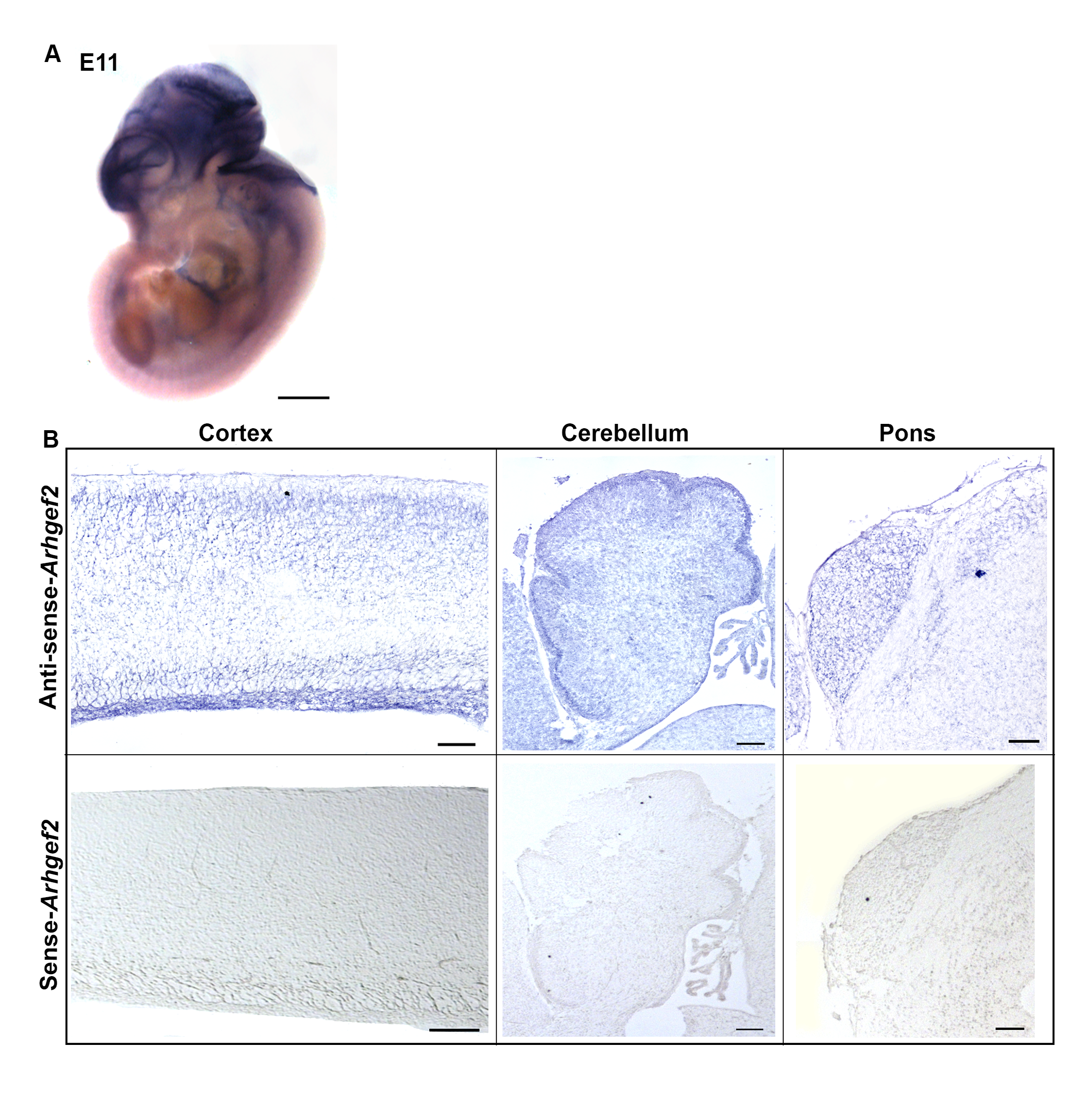

Supplement: S5 Fig — (A) Arhgef2 is strongly expressed in the neuroepithelium of the brain at E11. (B) At P0, Arhgef2 is expressed predominantly in the VZ/SVZ and upper layer of the neocortex, in the external granule layer (EGL) of the cerebellum and in the pontine nuclei (DIC images of in situ hybridization with anti-sense and sense Arhgef2 RNA probes, n = 4, scale bar 1 mm (A), scale bar 100 μm (B)). (TIF) [file pgen.1006746.s011.tif]

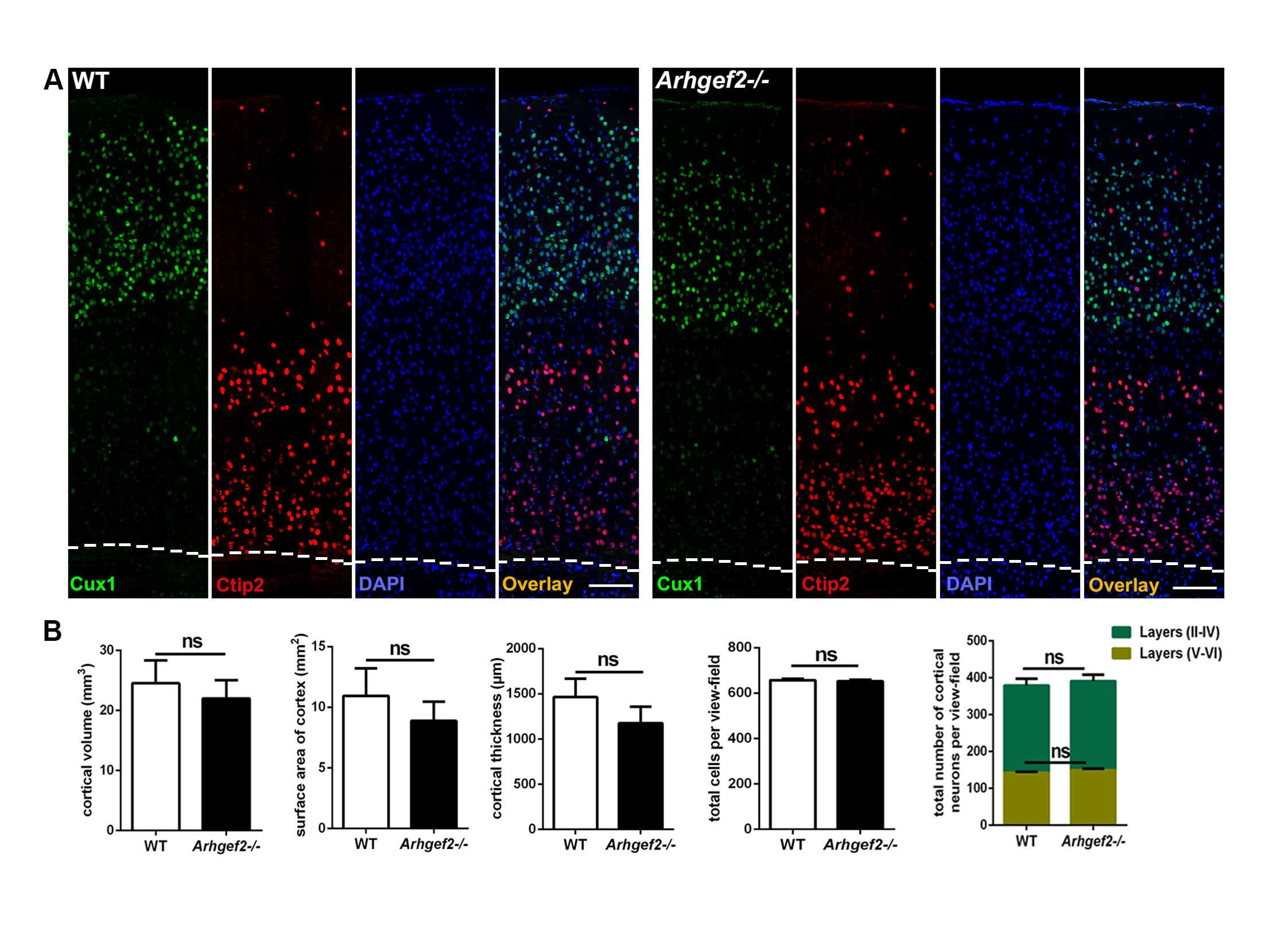

Supplement: S6 Fig — (A) Representative fluorescence micrographs of WT and Arhgef2-/- mice cortex stained for layers II-IV (Cux1, green), layers V-VI (Ctip2, red) and DAPI. (B). Quantification of cortical volume, surface area, thickness, total number of DAPI cells, Cux1 and Ctip2 positive cells per view field revealed no significant difference between control and mutant mice. (n = 3–4, Student’s t-test, ns- not significant, error bars represent ± SD, scale bar 100 μm). (TIF) [file pgen.1006746.s012.tif]

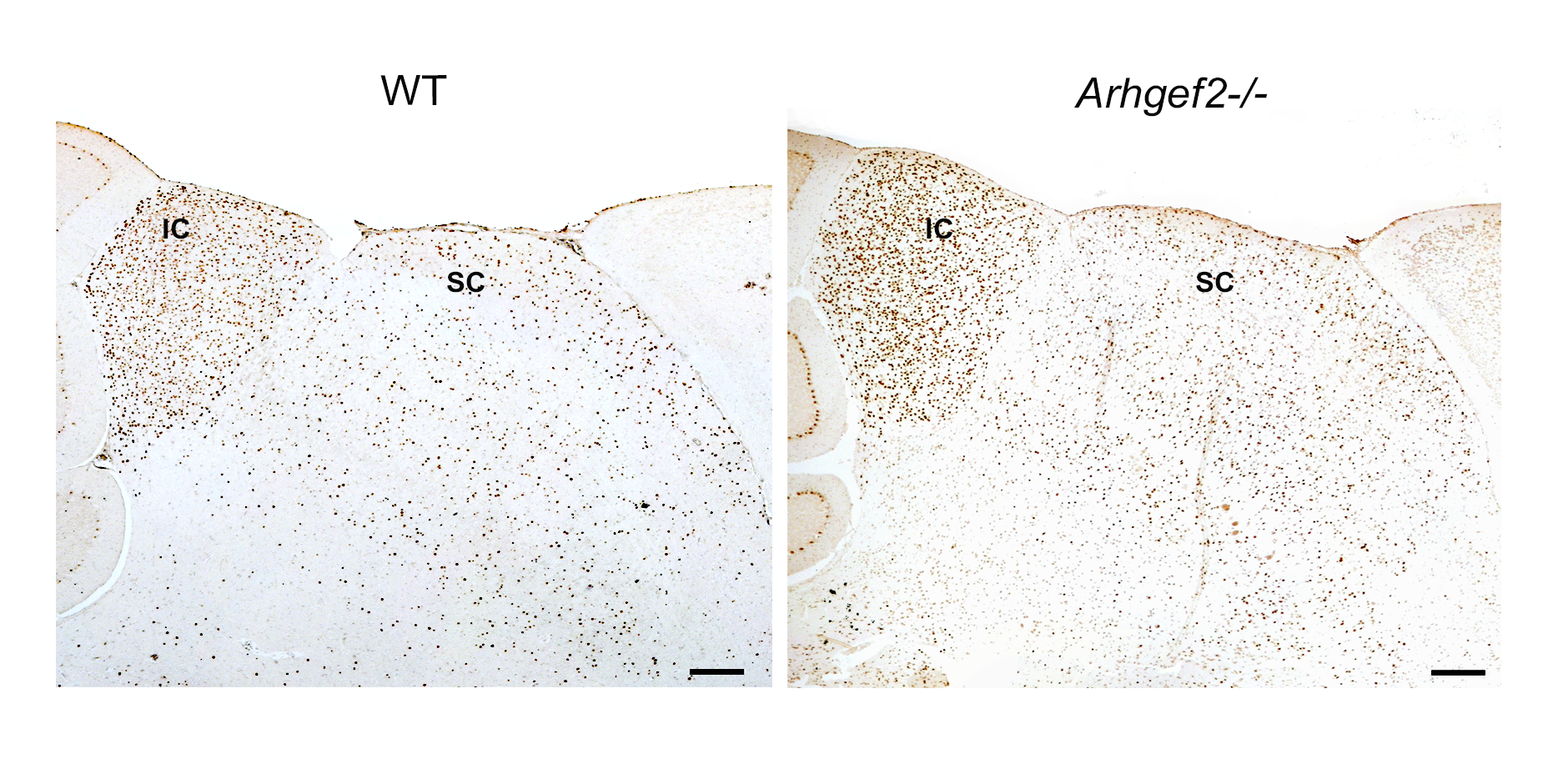

Supplement: S7 Fig — Representative DAB (3, 3'-Diaminobenzidine) stained micrographs of WT and Arhgef2-/- mice showing normal structures of FoxP2-positive inferior colliculus and superior colliculus. Scale bar 200 μm. (TIF) [file pgen.1006746.s013.tif]

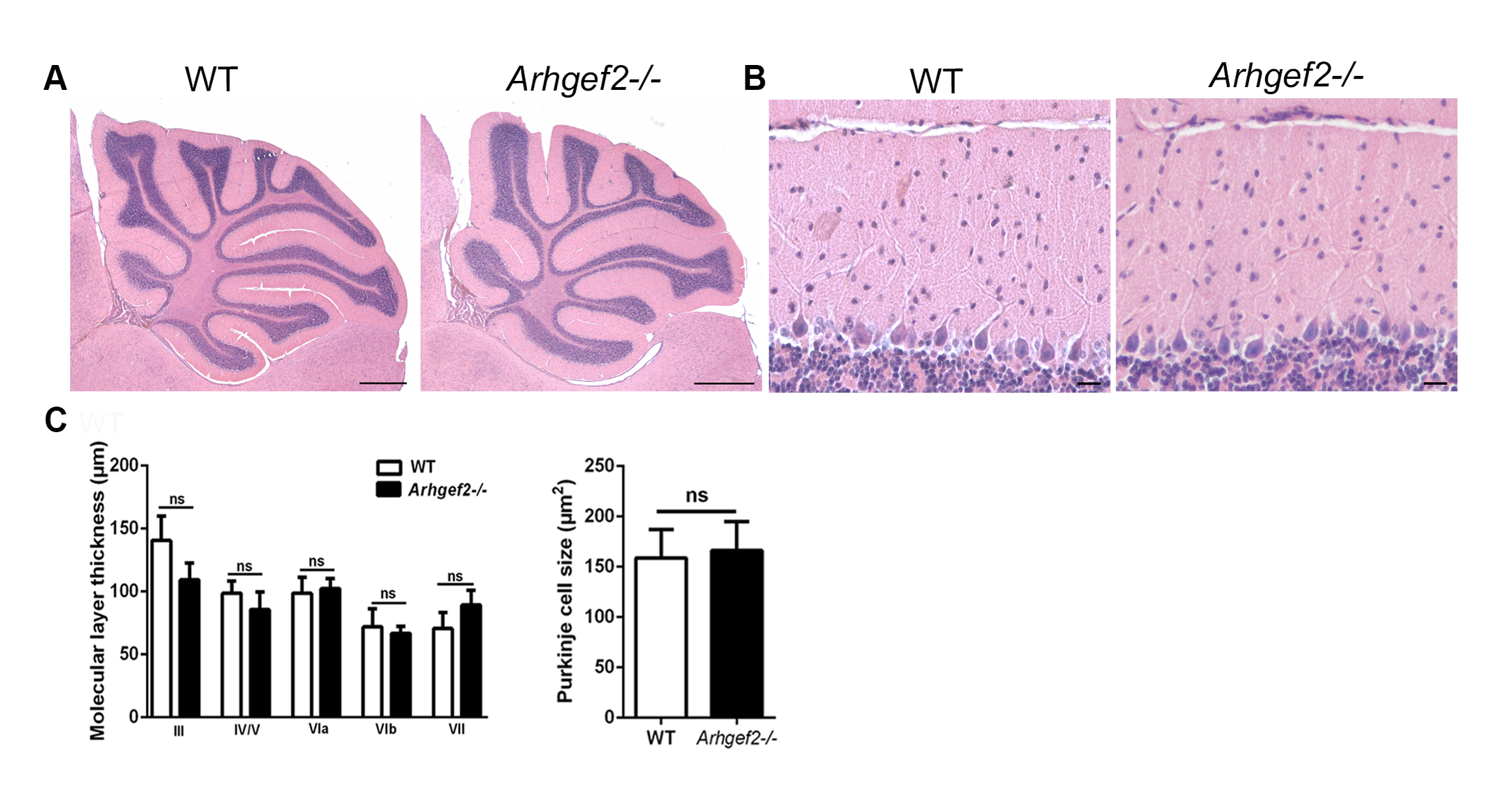

Supplement: S8 Fig — (A) Representative H&E stained micrographs of WT and mutant cerebellum (scale bar 500 μm), and (B) magnified cerebellar folia. (C) No significant change in Purkinje cell size and thickness of the molecular layer was observed in the mutant mice upon quantification. (n = 3–4, Student’s t-test, ns- not significant, error bars represent ± SD, scale bar 20 μm). (TIF) [file pgen.1006746.s014.tif]

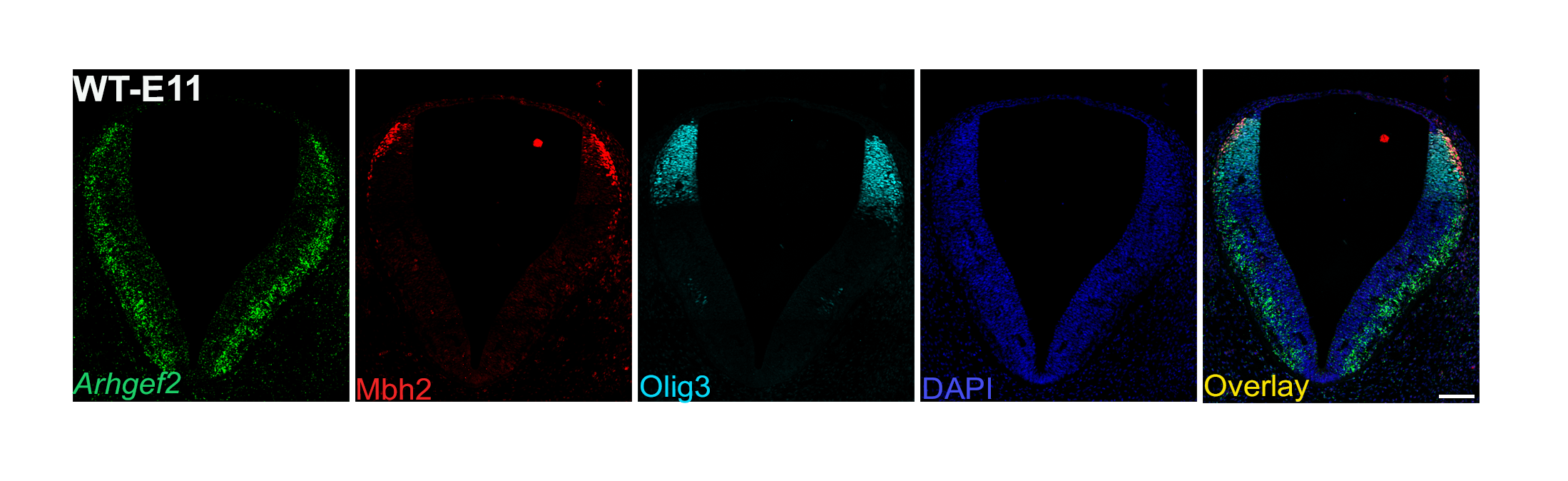

Supplement: S9 Fig — Photomicrographs representing transverse E11 section of dorsal hindbrain stained for Arhgef2 (green), Mbh2 (red), Olig3 (cyan), and DAPI, indicates a clear colocalisation in the mantle zone along migratory pathway. Scale bar 100 μm. (TIF) [file pgen.1006746.s015.tif]
